# GAPDH-A Recruits a Plant Virus Movement Protein to Cortical Virus Replication Complexes to Facilitate Viral Cell-to-Cell Movement

**DOI:** 10.1371/journal.ppat.1004505

**Published:** 2014-11-20

**Authors:** Masanori Kaido, Kazutomo Abe, Akira Mine, Kiwamu Hyodo, Takako Taniguchi, Hisaaki Taniguchi, Kazuyuki Mise, Tetsuro Okuno

**Affiliations:** 1 Laboratory of Plant Pathology, Graduate School of Agriculture, Kyoto University, Kyoto, Japan; 2 Institute for Enzyme Research, The University of Tokushima, Tokushima, Japan; University of Kentucky, United States of America

## Abstract

The formation of virus movement protein (MP)-containing punctate structures on the cortical endoplasmic reticulum is required for efficient intercellular movement of *Red clover necrotic mosaic virus* (RCNMV), a bipartite positive-strand RNA plant virus. We found that these cortical punctate structures constitute a viral replication complex (VRC) in addition to the previously reported aggregate structures that formed adjacent to the nucleus. We identified host proteins that interacted with RCNMV MP in virus-infected *Nicotiana benthamiana* leaves using a tandem affinity purification method followed by mass spectrometry. One of these host proteins was glyceraldehyde 3-phosphate dehydrogenase-A (NbGAPDH-A), which is a component of the Calvin-Benson cycle in chloroplasts. Virus-induced gene silencing of *NbGAPDH-A* reduced RCNMV multiplication in the inoculated leaves, but not in the single cells, thereby suggesting that *GAPDH-A* plays a positive role in cell-to-cell movement of RCNMV. The fusion protein of NbGAPDH-A and green fluorescent protein localized exclusively to the chloroplasts. In the presence of RCNMV RNA1, however, the protein localized to the cortical VRC as well as the chloroplasts. Bimolecular fluorescence complementation assay and GST pulldown assay confirmed *in vivo* and *in vitro* interactions, respectively, between the MP and NbGAPDH-A. Furthermore, gene silencing of *NbGAPDH-A* inhibited MP localization to the cortical VRC. We discuss the possible roles of NbGAPDH-A in the RCNMV movement process.

## Author Summary

Intercellular movement of plant viruses is the crucial step during systemic viral infections. *Red clover necrotic mosaic virus* (RCNMV), a bipartite positive-strand RNA plant virus, forms movement protein (MP)-containing punctate structures on the cortical endoplasmic reticulum in infected cells, which are required for efficient intercellular movement of the virus. We provide evidence that these cortical punctate structures constitute the viral replication complex (VRC), which forms during the early stage of virus infection. Furthermore, we show that a host protein of *Nicotiana benthamiana*, glyceraldehyde 3-phosphate dehydrogenase-A (NbGAPDH-A), possibly intercalates between the cortical VRC and MP. Knockdown of *NbGAPDH-A* diffused subcellular localization of MP and reduced intercellular movement of the virus. Chloroplastic NbGAPDH-A relocalized to the cortical VRC after infection with the virus. Our results suggest that the cortical VRC serves not only as the replication factory of viral RNA but also as a transportation hub, which transports viral RNA to neighboring uninfected cells via plasmodesmata.

## Introduction

Eukaryotic positive-strand RNA viruses replicate their genomes using membrane-bound virus replication complexes (VRC), which contain viral replicase proteins, viral RNA templates, and host-factor proteins [1]–[5]. Viral replicase proteins modify the host intracellular membrane morphology, including swelling, invagination, and the formation of spherules. Thus, VRC formation is accompanied by the remodeling of intracellular membranes [3]. Studies of the subcellular localization of viral proteins using immunoelectron microscopy or confocal laser scanning microscopy (CLSM) have shown that the movement proteins (MPs) of several plant viruses colocalize with the viral replicase protein [6]–[10], thereby suggesting that MPs are also components of VRCs. However, the MPs that localize to VRCs are not likely to be involved in the replication of viral genomic RNA because mutant viruses that do not encode functional MP can accumulate viral genomic RNA similar to that of the wild type virus with functional MP in infected protoplasts [11], [12]. MPs play central roles in the cell-to-cell and systemic movement of plant viruses, and they have been investigated intensively to determine their biochemical characteristics; their subcellular localization, including the cellular pathways that target them to the plasmodesmata (PD), the cytoplasmic channels connecting plant cells; and their interactions with host membranes or proteins [13]–[17].

The early process of VRC formation has been well characterized in *Tobacco mosaic virus* (TMV), which is the type member of the well-studied genus *Tobamovirus* [18]. TMV virions that enter host plant cells are partially uncoated, and the exposed 5′ cap structures are assumed to be recruited by unknown host factors to form small particles that contain viral RNA on the cortical endoplasmic reticulum (ER), before moving along the ER and actin filaments. The replication cycles starts after translation of the replicase proteins and the VRC increases in size. The punctate-like TMV VRC forms on the cortical ER and moves along actin filaments [18]–[20] or microtubules [21], [22]. The intracellular movement of VRC on actin filaments requires motor proteins such as myosin, and it is considered to be necessary for the targeting of viral genomic RNA to the PD [23]–[25]. The roles of TMV MP in the VRC are unknown. The “126-bodies”, which comprise the fusion protein of TMV 126-kDa replicase component protein and green fluorescent protein (GFP), can move along actin filaments without MP [20].

*Red clover necrotic mosaic virus* (RCNMV) is a positive-strand RNA virus with a bipartite genome that belongs to the genus *Dianthovirus*, in the family *Tombusviridae* [26]. Genomic RNA1 encodes p27 auxiliary replication protein, p88 RNA-dependent RNA polymerase (RdRp), and coat protein (CP), while RNA2 encodes MP (Figure S1A). p27 and p88 induce the production of an aggregate structure from ER membrane and they form the 480-kDa replication complex, which is a key enzyme complex for virus replication, via interactions with host chaperone proteins such as heat shock protein (HSP) 70 and HSP90, and membrane traffic-associated proteins such as Arf1 and Sar1 [27]–[31]. Using a bimolecular fluorescence complementation (BiFC) assay, these four host factors were shown to interact directly with p27 in the large aggregate adjacent to the nucleus in *Nicotiana benthamiana* cells [30], [31]. However, when the p27-GFP fusion protein was expressed with p88 and RNA2, it formed small punctate structures on the cortical ER and later formed large aggregates adjacent to the nucleus [32]. These results suggest that RCNMV VRC forms small punctate structures on the cortical ER, which then change their subcellular localization to form a large aggregate adjacent to the nucleus.

RCNMV MP belongs to the 30K superfamily and it is required for viral cell-to-cell and systemic movement [33], [34]. RCNMV is considered to pass through PD in the form of a viral RNA-MP complex because CP is dispensable for viral cell-to-cell movement [34] and MP also has the ability to bind single-stranded nucleic acids [35]. Microinjected RCNMV MP can increase the size-exclusion limit of PD and enable the transport of coinjected viral RNA into neighbor cells [36]. Alanine-scanning mutant analysis was used to determine the functional domains of the MP that bind RNA and that target it to PD, both of which are required for viral cell-to-cell movement [37], [38]. However, the cellular pathway that allows MP and/or MP-viral RNA complexes to target PD is unknown.

Previously, we reported the subcellular localization of the fusion protein of RCNMV MP and GFP (MP-GFP) in *N. benthamiana* [10]. In addition to PD localization, MP-GFP expressed by a recombinant virus formed punctate structures with p27 on the cortical ER. Transiently expressed MP-GFP also localized to punctate structures on the cortical ER, which was associated with the replication of RNA1, but not with that of RNA2. These results suggest that MP is recruited to the cortical ER by the viral replicase complexes formed with RNA1. To demonstrate the importance of cortical punctate structures containing MP, we conducted a deletion analysis of MP and showed that 70 C-terminus amino acids are required for both cortical punctate structure formation and viral cell-to-cell movement [39]. Based on these results, we hypothesized that the recruitment of MP by the viral replication complex might help MP to acquire viral genomic RNA1 that does not encode MP, thereby leading to the efficient cell-to-cell movement of RNA1.

To further investigate the mechanism that facilitates the movement of RCNMV, we performed tandem affinity purification of MP from virus-infected *N. benthamiana* leaves and analyzed the co-purified host proteins, by mass spectrometry. One of these host proteins was glyceraldehyde 3-phosphate dehydrogenase subunit A (GAPDH-A). GAPDHs are ubiquitous enzymes involved in glycolysis and gluconeogenesis, and GAPDH-A is a component of the Calvin-Benson cycle of photosynthetic organisms [40]. GAPDH-A and another subunit, GAPDH-B, are both located in the chloroplast in plants and algae [41]. Thus, we isolated the full-length cDNA of *N. benthamiana GAPDH-A* (*NbGAPDH-A*) and investigated its involvement in RCNMV multiplication. Our results demonstrate that NbGAPDH-A is involved in virus cell-to-cell movement by influencing MP localization to the VRC. We discuss the possible mechanism that underlies this process.

## Results

In the course of this study, we used many recombinant RCNMVs listed in Figure S1. Fluorescent proteins (FPs) or fusion proteins of MP with FP were expressed from the recombinant RNA1, in which the *CP* gene had been replaced. In those cases RNA2 or MP-frameshifted RNA2 were included in the inocula, because the stem-loop structure in RNA2 is required for the transcription of CP-subgenomic RNA from RNA1 [42].

### Cortical punctate structures containing RCNMV MP are the sites of viral RNA replication

Previously, we reported that RCNMV MP colocalized with the viral replicase protein p27 to the punctate structures on the cortical ER in virus-infected *N. benthamiana* cells during the early stage of infection. Later, most of these cortical punctates disappeared and a large aggregate was formed adjacent to the nucleus in epidermal cells [10], [32]. These aggregates contained newly synthesized viral RNAs and the host-factor proteins essential for replication, and they were shown to be the sites of RCNMV RNA replication [30], [31]. However, no evidence of viral RNA replication in the cortical punctates has been reported. Thus, we detected double-stranded RNA (dsRNA), the replication intermediates of positive-stranded RNA viruses, by immunostaining using the antibody against double-stranded RNA (J2 antibody) in *N. benthamiana* protoplasts. J2 antibody has been widely used to detect the replication sites of animal and plant RNA viruses, and cellular RNAs such as ribosomal RNA are below the limit of detection [43]–[45]. *N. benthamiana* protoplasts were inoculated with *in vitro* transcripts of recombinant RCNMV, which expressed the fusion protein of MP and a red FP, mCherry (MP-mCherry, Figure S1B).

**Figure 1:**
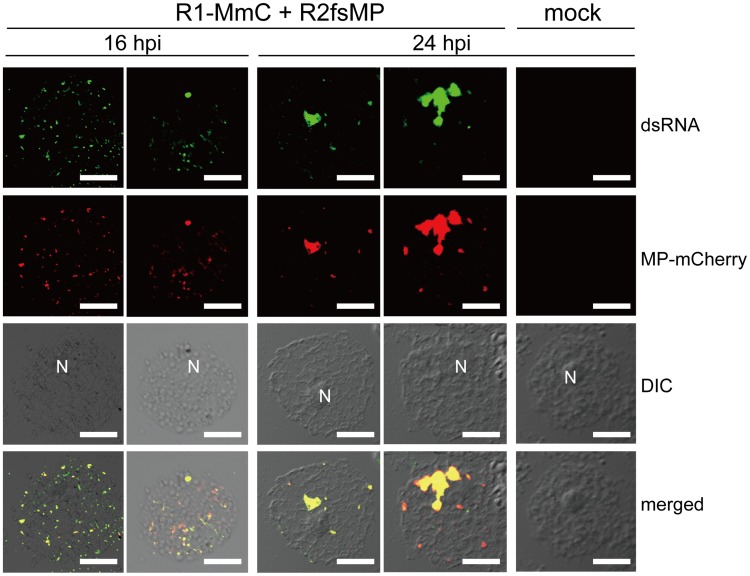
Cortical punctate structures that contain RCNMV MP are sites of viral RNA replication. *Nicotiana benthamiana* protoplasts were inoculated with recombinant RCNMV RNAs that expressed the MP-mCherry fusion protein (Figure S1B) and subjected to immunostaining with anti-dsRNA primary antibody followed by Alexa Fluor 488-conjugated secondary antibody at an early stage of infection (16 hpi, left 2 rows of panels) and at a late stage of infection (24 hpi, center 2 rows of panels). The right-most panels show the results for mock-inoculated protoplasts treated with the same antibodies. Images present confocal projections of five optical sections at 1 µm intervals, which range from the surface to the middle of the protoplasts. DIC: differential interference contrast, N: nucleus. Scale bar = 20 µm.

Using CLSM, MP-mCherry and dsRNA were detected as overlapping small punctate signals near the surfaces of protoplasts at 16 h post inoculation (hpi) (Figure 1, left 2 rows of panels). At 24 hpi, most of these small punctates disappeared and large aggregates were detected adjacent to the nucleus, which contained both MP and dsRNA (Figure 1, center 2 rows of panels), thereby confirming the results reported previously [30], [31]. No fluorescent signals for dsRNA were detected in mock-inoculated protoplasts (Figure 1, right panels). These results indicate that both the cortical punctates formed during an early stage of RCNMV infection and the aggregates formed adjacent to the nucleus during the later stage of infection are the sites of RCNMV RNA replication. Subsequently, we refer to the small punctate-like structures that contain the MP in the cortical region as ‘cortical VRC.’

### Identification of host plant proteins that interact with RCNMV MP

To identify the host proteins that interact with RCNMV MP, we performed two-step affinity purification of MP fused to a tandem affinity purification tag sequence. The tagged MP was functional because it supported virus cell-to-cell and systemic movement with the same efficiency as the native MP in *N. benthamiana* plants (Figure S2).

**Figure 2:**
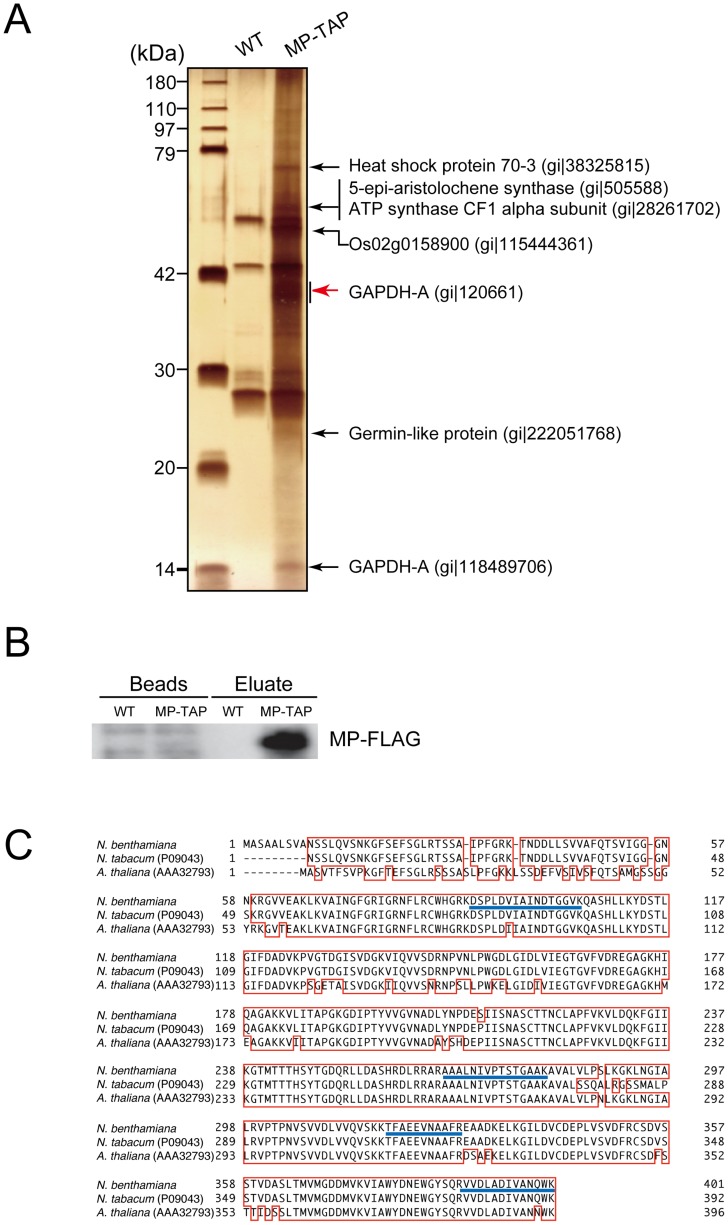
Identification of proteins that were copurified with RCNMV MP. Protein extracts prepared from *Agrobacterium*-infiltrated leaves that expressed RCNMV RNA1 plus RNA2 (WT, pBICR12, Figure S1C), or RNA1 plus recombinant RNA2 encoding MP tagged with TAP tag sequences (MP-TAP, pBICR12/MP-TAP, Figure S1D), were subjected to two-step affinity purification using an anti-HA antibody followed by an anti-FLAG antibody. (A) One fifth of the affinity-purified fractions eluted from the anti-FLAG beads (Eluate) and the proteins contained with the beads after elution (Beads) were subjected to Western blotting using anti-FLAG antibody. (B) Four fifths of the affinity-purified fractions were subjected to SDS-PAGE and stained using MS-compatible silver staining. The protein bands of interests were excised, subjected to in-gel digestion, and analyzed by tandem mass spectrometry. Proteins with Mascot search scores >50, which were absent from the control protein bands, and proteins with significantly higher scores than the control proteins are indicated on the right-hand sides of the panels. The NCBI accession numbers of the identified proteins are also indicated. (C) The *GAPDH-A* gene of *N. benthamiana* was cloned and the deduced amino acid sequence was aligned using GENETYX-Mac ver. 14.0.1 with its closest homolog from *N. tabacum* (accession number P09043) and that from *Arabidopsis thaliana* (accession number AAA32793). The blue lines show the peptide sequences detected.

Binary vector plasmid pBICR12/MP-TAP (Figure S1D), and pBICR12 (Figure S1C) as the negative control, were infiltrated via *Agrobacterium* into *N. benthamiana*. The tandem affinity purified fraction prepared from pBICR12/MP-TAP-infiltrated leaves contained several silver-stained bands, which were not detected in the negative control (Figure 2A). The clear silver-stained band that represented the MP-FLAG was not detected in the MP-TAP lane for unknown reason. Considering its size (35.6 kDa), the band is probably masked in the broad range of the stained area below the 42 kDa marker. Actually nano-liquid chromatography-tandem mass spectrometry (LC/MS/MS) analysis demonstrated that a piece of wide gel cut out from MP-TAP lane (Figure 2A, red arrow) contained the MP (Table S1). MP-FLAG was also detected by Western blotting analysis in the tandem affinity purified fraction prepared from pBICR12/MP-TAP-infiltrated leaves but not from the negative control leaves (Figure 2B).

These silver-stained bands in the MP-TAP lane, and the similar regions of the gel for the negative control lane were excised and subjected to in-gel trypsin digestions and LC/MS/MS analyses. We identified RCNMV MP and several host proteins from the stained bands, and these proteins were not detected from the negative control gels. Among these, we focused on GAPDH-A. A partial *GAPDH-A* sequence was amplified by RT-PCR using the total RNA of *N. benthamiana*, where the primer designs were based on *N. tabacum GAPDH-A*. The full-length cDNA of *GAPDH-A* was cloned according to 5′ and 3′ RACE methods, which we refer to as *NbGAPDH-A* (accession number AB937979). The deduced amino acid sequence of *NbGAPDH-A* was almost identical to the reported partial *GAPDH-A* of *N. tabacum* (96.9% shared identity, except for nine N terminal amino acids) and very similar to that of *Arabidopsis thaliana*, except for 60 N terminal amino acids (Figure 2C).

### VIGS of *NbGAPDH-A* negatively affects the multiplication of RCNMV

**Figure 3:**
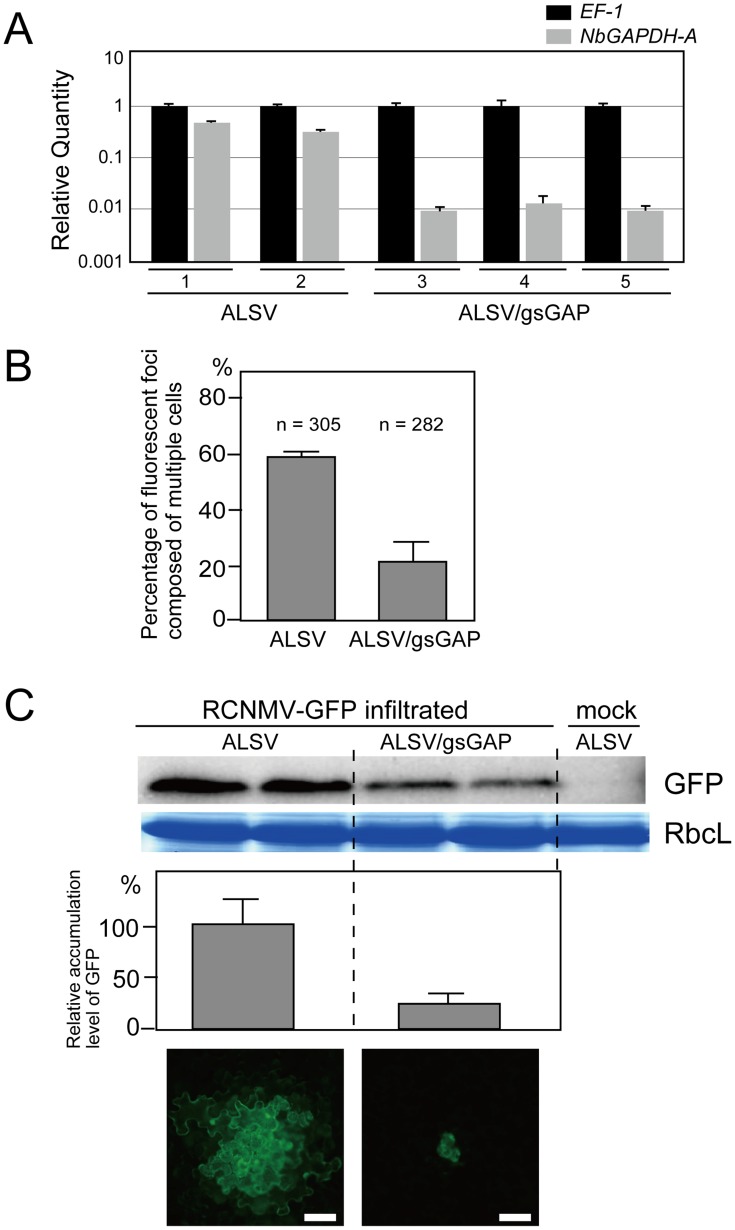
Multiplication of RCNMV is inhibited in *NbGAPDH-A*-silenced *N. benthamiana* leaves. (**A**) Gene silencing of *NbGAPDH-A* was induced in *N. benthamiana* plants inoculated with the ALSV vector, which harbored a 294 bp partial fragment (nucleotides 352–645 from start codon) of *NbGAPDH-A* (ALSV/gsGAP), via *Agrobacterium*. The empty ALSV vector (ALSV) was used as a control. Total RNA was prepared from two ALSV-infected and three ALSV/gsGAP-infected plants. *NbGAPDH-A* mRNA levels were determined by real time PCR using primers specific to *NbGAPDH-A* (nucleotides 755–954 from start codon). The real time PCR results for the *EF-1* mRNA (closed column) were used to adjust the relative accumulation levels of *NbGAPDH-A* mRNA (gray column). (**B**) *In vitro* transcripts of a recombinant RCNMV that expressed GFP from its subgenomic RNA (RCNMV-GFP, Figure S1E) were inoculated mechanically onto ALSV- or ALSV/gsGAP-infected *N. benthamiana* plants. At 20 hpi, the percentages of fluorescent foci that comprised multiple cells were measured using epifluorescence microscopy. ‘*n*’ represents the total number of fluorescent foci in 4 inoculated leaves (about 25 square centimeters). (**C**) An *Agrobacterium* culture that contained the pBICR1sG2 plasmid, which expressed RCNMV-GFP (Figure S1F), was diluted to OD_600_ = 0.03 and infiltrated into ALSV- or ALSV/gsGAP-infected *N. benthamiana* plants. At 35 hpi, protein was extracted from the infiltrated leaves and subjected to Western blotting using anti-GFP antibody. RbcL is a Coomassie brilliant blue-stained gel image, which shows the large subunit of Rubisco proteins. The accumulated levels of GFP from three separate experiments were quantified using the Image Gauge program and plotted in the graph. The lowest two panels show representative epifluorescence microscopy images of the infiltrated leaves at 35 hpi. Scale bar = 50 µm.

To investigate the possible involvement of *NbGAPDH-A* in RCNMV multiplication, we downregulated the gene using the *Apple latent spherical virus* (ALSV) vector [46]. A plasmid that expressed wild type ALSV, or that expressed the recombinant ALSV containing 294 nucleotides of *NbGAPDH-A* (ALSV/gsGAP vector), was mobilized into *Agrobacterium* and the bacterium was used to inoculate young *N. benthamiana* plants. The accumulation level of *NbGAPDH-A* mRNA in the newly developed leaves was determined 2–3 weeks later by real time RT-PCR. *NbGAPDH-A* was silenced effectively in ALSV/gsGAP vector-infected plants; the mRNA level of *NbGAPDH-A* was reduced to 3% of that in the wild type ALSV-infected plants (Figure 3A). This result coincided with that by semi-quantitative RT-PCR in which mRNA level in ALSV/gsGAP vector-infected plants was about 1/32 of that in wild type ALSV-infected plants (Figure S3). No symptoms or growth inhibition were detected in the *NbGAPDH-A*-silenced plants and wild type ALSV-infected plants (Figure S4, see Discussion). Hereafter, all of the ALSV/gsGAP-infected plants and the protoplasts prepared from those plants were tested by real time or semi-quantitative RT-PCR to confirm the *NbGAPDH-A* gene was silenced.

We then subjected the ALSV- and ALSV/gsGAP-infected plants to challenge via the mechanical inoculation of *in vitro* transcripts of the recombinant RCNMV containing the GFP gene (RCNMV-GFP; Figure S1E). The percentage of fluorescent foci with multiple cells in the ALSV/gsGAP-infected plants was about 1/3 of that in the ALSV-infected plants at 20 hpi (Figure 3B). The result suggests that RCNMV multiplication was negatively affected by the silencing of *NbGAPDH-A*.

In order to evaluate the effect of the gene silencing on RCNMV multiplication more objectively, we further performed challenge inoculation with pBICR1sG2 (Figure S1F), which expressed RCNMV-GFP, via *Agrobacterium* infiltration, and the multiplication level of the recombinant virus was estimated by western blot analysis for GFP. The level of GFP accumulation at 35 hpi in the leaves of ALSV/gsGAP-infected plants was approximately 20% of that in the leaves of the ALSV-infected plants (Figure 3C). The majority of the fluorescent foci were comprised of more than 10 cells in the latter plants, whereas such a wide spread of fluorescence was barely detected in the former plants (Figure 3C, lower panels). At 48 hpi, most of the fluorescent foci in the ALSV/gsGAP-infected plants became larger and the level of GFP accumulation was about 80% of that in the wild type ALSV-infected plants (Figure S5). Thus, RCNMV multiplication was impaired in the *NbGAPDH-A*-silenced *N. benthamiana* leaves, at an early stage of infection.

To investigate whether downregulation of the *NbGAPDH-A* gene could affect the multiplication of viruses other than RCNMV, we inoculated ALSV- and ALSV/gsGAP-infected *N. benthamiana* plants with a recombinant *Tomato mosaic virus* (ToMV), where the *CP* gene was replaced with the *GFP* gene. The spread of GFP fluorescence was indistinguishable at 40 and 48 hpi by epifluorescence microscopy and the GFP accumulation level was also similar in both plants (Figure S6). These results indicate that the *NbGAPDH-A* gene is not involved in the multiplication of ToMV.

### VIGS of *NbGAPDH-A* does not affect RCNMV replication

**Figure 4:**
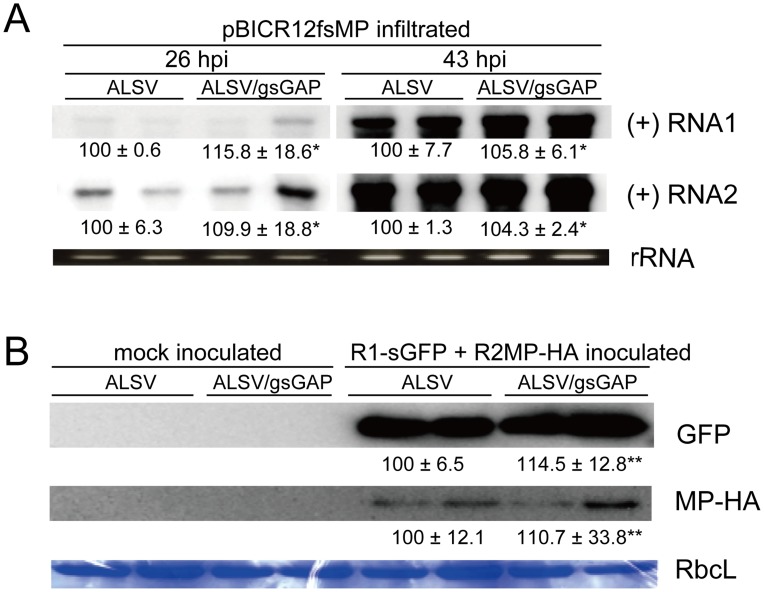
*NbGAPDH-A* does not affect the accumulations of RCNMV RNA and MP at the single-cell level. (**A**) An *Agrobacterium* culture that contained the pBICR12fsMP plasmid, that expressed movement-deficient RCNMV (Figure S1G), was diluted to OD_600_ = 0.8 and infiltrated into ALSV- or ALSV/gsGAP-infected *N. benthamiana* plants (two plants for each). At 26 hpi and 43 hpi, the total RNA was extracted from the infiltrated leaves and subjected to Northern blotting using DIG-labeled riboprobes specific for the plus-strand (+) RNA1 or RNA2 of RCNMV. rRNA is an ethidium bromide-stained agarose gel image of 1 µg total RNA, which was used as the loading control. The numbers below the images represent the relative accumulation levels (means ± SE) of viral RNAs using the Image Gauge program (Fuji Film), which were calculated based on two independent experiments. Asterisk indicates a not significant (P>0.05; Student's *t*-test) difference compared with the viral RNA accumulation level in the protoplasts from ALSV-infected *N. benthamiana*. (**B**) Protoplasts prepared from ALSV- or ALSV/gsGAP-infected *N. benthamiana* plants (each two plants) were inoculated with a mixture of *in vitro* transcripts of the recombinant RCNMV RNA1, which expressed GFP from subgenomic RNA; and RNA2, which expressed MP tagged with HA (Figure S1H). Proteins extracted from 2×10^4^ protoplasts were loaded in each lane. GFP and MP-HA were detected using a rabbit polyclonal antibody against GFP and a rat polyclonal antibody against HA, respectively. RbcL is a Coomassie brilliant blue-stained gel image of proteins extracted from 2×10^4^ protoplasts, which shows the large subunit of Rubisco proteins. The numbers below the images represent the relative accumulation levels (means ± SE) of the proteins using the Image Gauge program (Fuji Film), which were calculated from two independent experiments. Double asterisk indicates a not significant (P>0.05; Student's *t*-test) difference compared with the protein accumulation level in the protoplasts from ALSV-infected *N. benthamiana*.

To investigate the effect of *NbGAPDH-A* silencing on RCNMV accumulation at the single cell level, we infiltrated ALSV-, or ALSV/gsGAP-infected plants with *Agrobacterium* that contained pBICR12fsMP, which expressed movement-deficient RCNMV RNAs (Fig. S1G) [10]. At 26 and 43 hpi, similar amounts of positive-stranded viral RNAs accumulated (Figure 4A), thereby suggesting that RCNMV multiplied at similar levels in the initially infected cells.

To further investigate the multiplication levels of RCNMV in single cells, protoplasts were prepared from ALSV- and ALSV/gsGAP-infected plants and inoculated with *in vitro* transcripts of the recombinant RCNMV, which expressed GFP and MP tagged with HA (MP-HA) (Figure S1H). Similar amounts of GFP accumulated in both protoplasts (Figure 4B, upper panel), thereby indicating that gene silencing of *NbGAPDH-A* did not affect the accumulation of the recombinant virus at the single cell level. We also analyzed the accumulation of MP-HA. As shown in the middle panel of Figure 4B, the levels of MP-HA were similar with either inoculation, which suggests that NbGAPDH-A is not involved in the translational control or stability of MP. Overall, these results suggest that NbGAPDH-A is unlikely to be involved in the replication of RCNMV RNAs and that it is involved in the cell-to-cell movement of RCNMV via its interaction with MP.

### Subcellular localization of NbGAPDH-A

**Figure 5:**
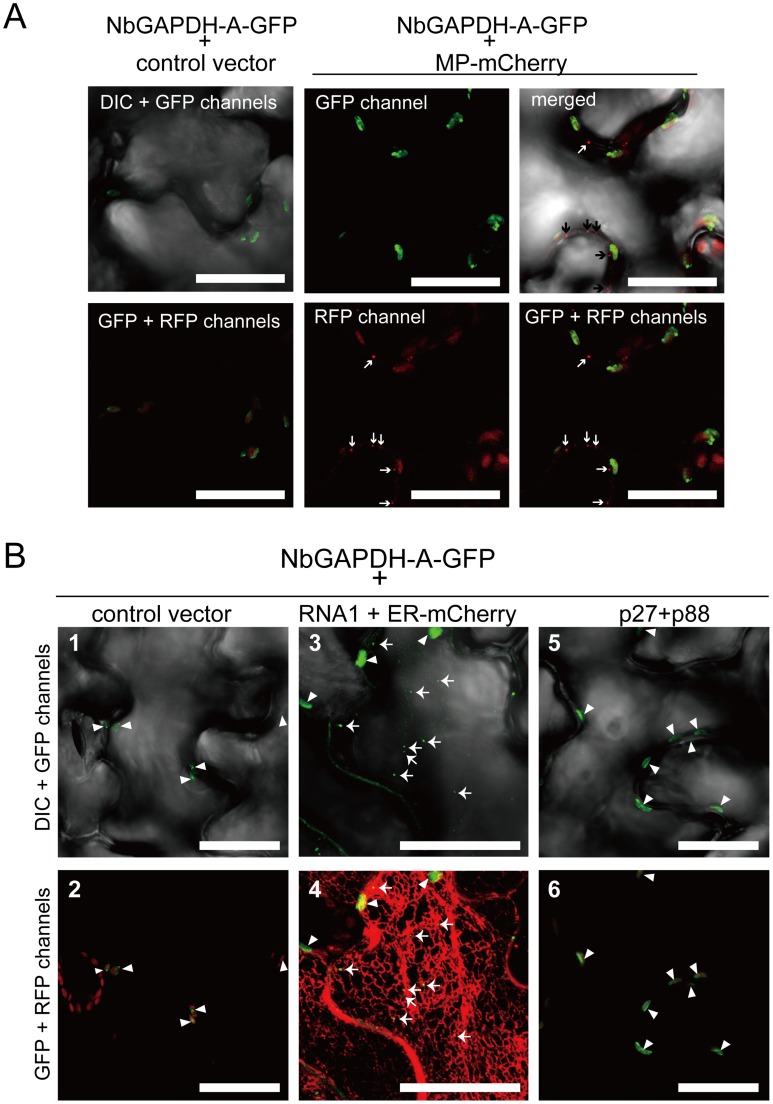
NbGAPDH-A changes its subcellular localization in association with RCNMV RNA replication, but not with viral proteins. Representative CLSM images of agroinfiltrated *N. benthamiana* epidermal cells. Each *Agrobacterium* culture was diluted to OD_600_ = 0.8 and equal volume of cultures were mixed for infiltration. Confocal microscopy images were taken at 40 h after infiltration. Images present confocal projections composed of 15 optical sections taken at 1 µm intervals, from the surface to the middle of epidermal cells. Chloroplast autofluorescence was detected in all the RFP-channel images by the use of 610IF emission filter. Scale bar = 20 µm. (**A**) Epidermal cells transiently expressing NbGAPDH-A-GFP (left two panels), and NbGAPDH-A-GFP with MP-mCherry (right four panels). Arrows represent PD-localized MP-mCherry signals. (**B**) Epidermal cells, which transiently expressed NbGAPDH-A-GFP (panels 1 and 2), or NbGAPDH-A-GFP with RCNMV RNA1 and ER marker (ER-mCherry) (panels 3 and 4), or NbGAPDH-A-GFP with viral replicase component proteins p27 and p88 (panels 5 and 6). Arrowheads represent chloroplast-localized NbGAPDH-A-GFP signals and arrows represent cortical ER-localized NbGAPDH-A-GFP signals.

NbGAPDH-A is assumed to localize to the chloroplasts. However, RCNMV replication occurs in association with the ER membrane and no relationship with the chloroplasts has been reported previously. To investigate the possible interaction between NbGAPDH-A and RCNMV proteins *in vivo*, we examined the subcellular localization of NbGAPDH-A in the absence or presence of RCNMV factors. When NbGAPDH-A tagged with GFP (NbGAPDH-A-GFP) alone was expressed transiently in *N. benthamiana* leaves via agroinfiltration, the protein localized exclusively to chloroplasts (Figure 5A, left two panels and Figure 5B, panels 1 and 2). The localization pattern of NbGAPDH-A-GFP was not altered by coexpression with RCNMV MP-mCherry. NbGAPDH-A-GFP signals were detected in the chloroplasts and were never detected in PD (Figure 5A, right four panels). This suggests that the transiently expressed MP does not interact with NbGAPDH-A *in vivo*.

**Figure 6:**
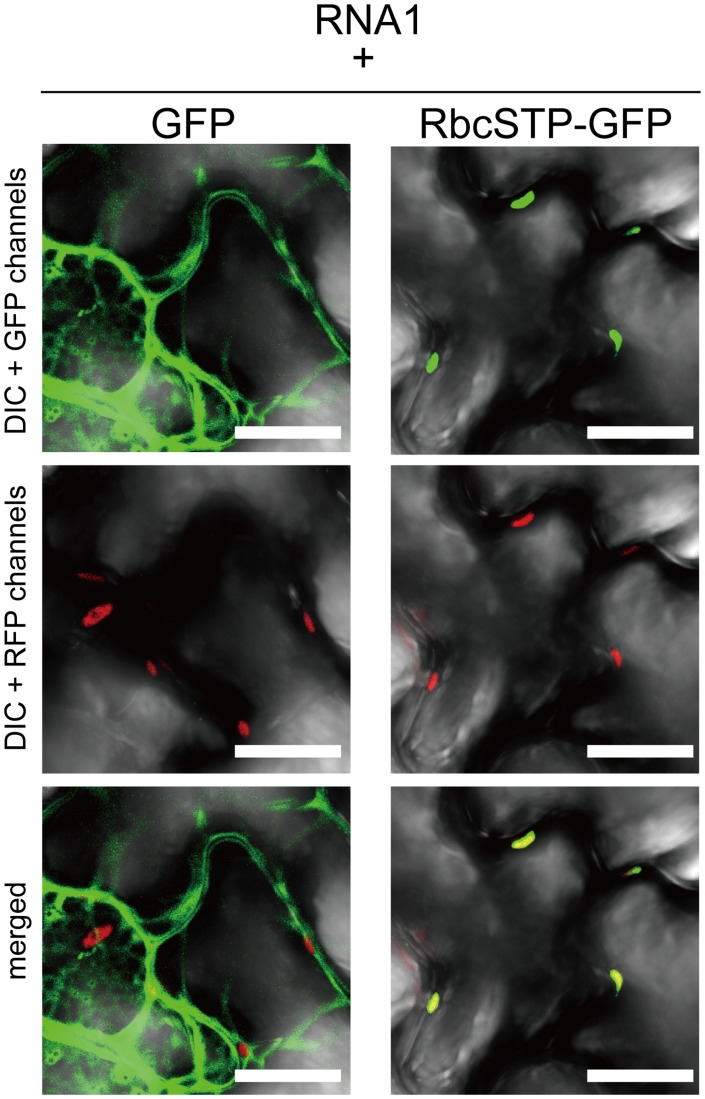
Subcellular localization of free GFP and GFP with chloroplast targeting signal is not affected by coexpression of RCNMV RNA1. Representative CLSM images of agroinfiltrated *N. benthamiana* epidermal cells. Epidermal cells transiently expressing RCNMV RNA1 together with GFP (left panels), or with GFP with chloroplast targeting signal peptide of Rubisco small subunit (RbcSTP-GFP, right panels). Scale bar = 20 µm. Conditions for infiltration, plant incubation, and CLSM were similar to those in Figure 5.

Next to investigate whether the subcellular localization of NbGAPDH-A-GFP could be affected by RCNMV RNA replication, NbGAPDH-A-GFP was coexpressed with RNA1. The GFP signals were detected in punctate structures that formed near the surface regions of epidermal cells, as well as in chloroplasts (Figure 5B, panel 3). These cortical signals colocalized with ER marker signals (Figure 5B, panel 4). Similar cortical punctate signals of NbGAPDH-A-GFP were also detected when it was coexpressed with both RNA1 and RNA2 (Figure S7), but not with the viral replicase component proteins, p27 and p88 (Figure 5B, panels 5 and 6). Lack of the cortical punctate signals of NbGAPDH-A-GFP in the latter leaves does not seem to be due to the low level of viral replicase proteins. p27 accumulated efficiently in the latter leaves (Figure S8). p88 was below the limit of detection in these agroinfiltrated leaves, as described previously [28], [29]. These results suggested the association between the localization of NbGAPDH-A-GFP to punctates on the cortical ER and the replication of RNA1. To examine this association is specific to NbGAPDH-A-GFP, we investigated the localization of free GFP or the GFP with chloroplast-targeting signal peptide in the presence of RNA1. No cortical punctate signals were detected in the leaves expressing these GFP proteins with RNA1 (Figure 6 and Figure S9). These results suggested that NbGAPDH-A was recruited to the cortical punctate structures in association with the replication of RNA1.

### NbGAPDH-A interacts with RCNMV MP *in vivo*

**Figure 7:**
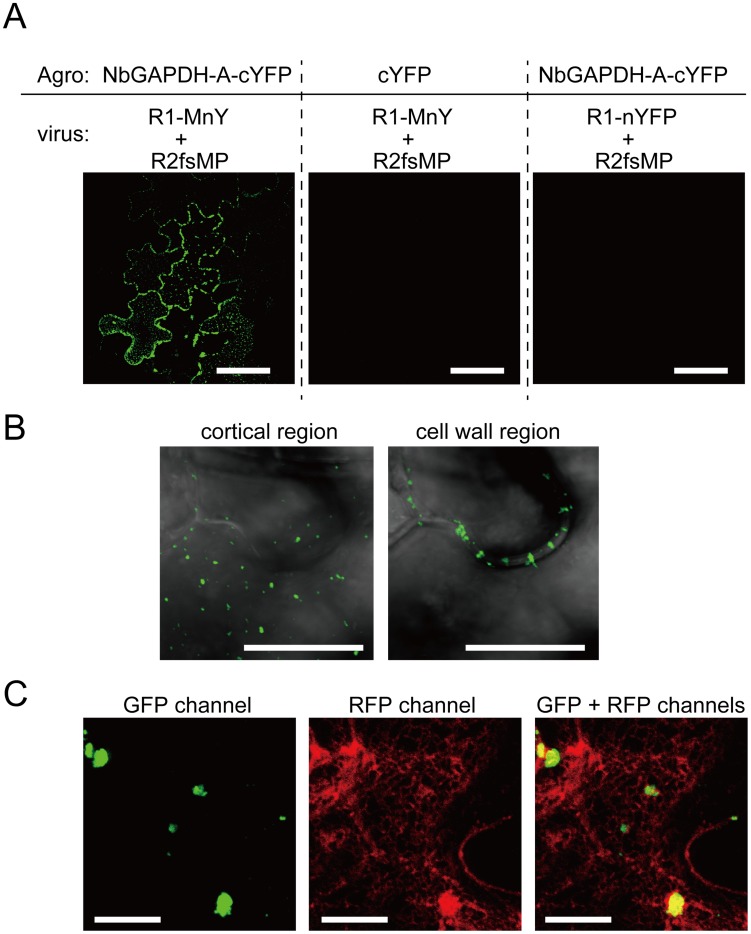
Bimolecular fluorescence complementation analyses of the interaction between RCNMV MP and NbGAPDH-A. NbGAPDH-A fused to the C-terminal half of YFP at the C-terminus (NbGAPDH-A-cYFP), or C-terminal half of YFP (cYFP) as the negative control, was expressed with TBSV silencing suppressor p19 in *N. benthamiana* leaves via *Agrobacterium* infiltration. 18 h after infiltration, *in vitro* transcripts of the recombinant RCNMV that expressed fusion protein of the MP and N-terminal half of YFP at the C-terminus (R1-MnY+R2fsMP, Figure S1I), or the recombinant virus that expressed N-terminal half of YFP (R1-nYFP+R2fsMP, Figure S1J) as the negative control, was mechanically inoculated. At 28 hpi of the recombinant virus, reconstructed YFP signal was visualized using CLSM. (**A**) Reconstituted YFP signals were detected as foci composed of 5–10 cells in the leaves that expressed NbGAPDH-A-cYFP and inoculated with R1-MnY+R2fsMP (left panel). No YFP signals were detected in the leaves that expressed unfusedcYFP (center panel), or those inoculated with R1-nYFP+R2fsMP (right panel). Scale bar = 50 µm (**B**) Large magnification images of the reconstituted YFP in the cortical (left panel) and inner cell wall region (right panel). Images are from optical sections taken at upper part for cortical region and middle part for cell wall region in the same site of the leaf and mergers of DIC and GFP channel. Scale bar = 10 µm (**C**) Reconstituted YFP signals as cortical punctates (left panel), ER-mCherry signals (center panel) and overlapped (right panel). Most YFP-punctates are larger than those in (B), because this cell is closer to the center of infection and that shows the cell is at a later stage of virus infection. Scale bar = 10 µm.

The interaction between NbGAPDH-A and RCNMV MP *in vivo* was confirmed by BiFC assays in *N. benthamiana* epidermal cells. NbGAPDH-A was fused to the C-terminal half of yellow fluorescent protein (YFP) at the C terminus (NbGAPDH-A-cYFP) and was expressed with *Tomato bushy stunt virus* (TBSV) silencing suppressor p19 in *N. benthamiana* via agroinfiltration. Recombinant RCNMV transcripts that expressed the MP fused to the N-terminal half of YFP at the C terminus (MP-nYFP, Figure S1I) was mechanically inoculated at 16 h post infiltration. At 28 hpi with the recombinant virus, fluorescence was observed using CLSM. YFP fluorescence was reconstituted in the presence of NbGAPDH-A-cYFP and the MP-nYFP (Figure 7A, left panel). No YFP fluorescence was detected in control experiments (Figure 7A, center and right panels; Figure S1J). With higher magnification, reconstituted YFP signals were observed as punctate structures in the cortical region (Figure 7B, left panel) and were also detected in the cell wall (Figure 7B, right panel, see Discussion). Reconstituted YFP signals in the cortical punctates were confirmed to overlap with ER marker signals (Figure 7C). These results, together with the localization results of the MP expressed from recombinant virus to the cortical VRC ([10] and Figure 1) show that the reconstituted YFP signals are on the cortical VRC.

### NbGAPDH-A interacts with RCNMV MP and p27 *in vitro*

**Figure 8:**
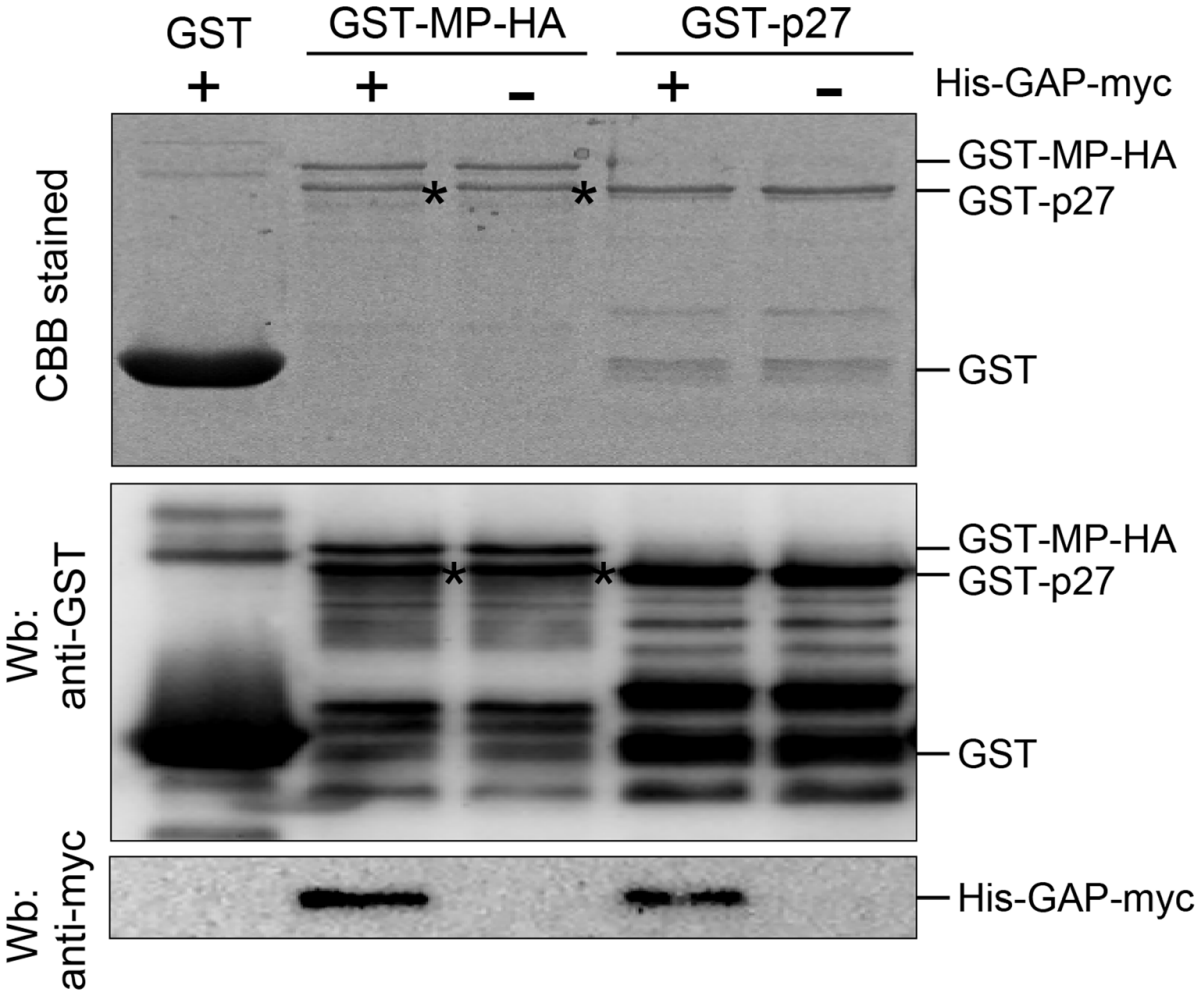
NbGAPDH-A interacts with both RCNMV MP and p27 *in vitro*. Glutathione resin-bound GST, GST-fused MP (GST-MP-HA) and GST-fused p27 (GST-p27) was incubated with the purified recombinant NbGAPDH-A (His-GAP-myc). After washing, the pulled-down complexes were subjected to SDS-PAGE and analyzed by Western blotting (Wb) using anti-GST and anti-myc antibodies. Same samples were subjected to SDS-PAGE and stained with Coomasie brilliant blue CBB). The bands that appeared in GST-MP-HA lanes (*) with almost similar mobility as GST-p27 are probably the degradation product of GST-MP-HA.

Subcellular localization results (Figure 5) and BiFC results (Figure 7) suggest that NbGAPDH-A interacts with both viral replicase protein(s) and MP in association with the replication of viral RNA. To confirm the direct interaction between NbGAPDH-A and RCNMV MP, or NbGAPDH-A and p27, we performed GST pulldown assays *in vitro*. Bacterially expressed and purified NbGAPDH-A with an N-terminal 6× His tag and C-terminal myc tag (His-GAP-myc) was incubated with N-terminally GST- and C-terminally HA-tagged MP (GST-MP-HA), N-terminally GST-fused p27 (GST-p27), or GST, which were captured on glutathione-bound beads. Immunoblot analyses using an anti-myc antibody demonstrated that His-GAP-myc was pulled down by GST-MP-HA and GST-p27, but not by GST (Figure 8), thereby indicating that His-GAP-myc binds to both MP and p27 *in vitro*. To rule out the possibility that coprecipitation in the GST-pulldown experiment was mediated by interaction with any unspecific RNA that bound to MP or p27, we included RNaseA to the reaction. Addition of 50 µg/ml of RNaseA did not affect the result (Figure S10), suggesting that NbGAPDH-A interacted with the MP and p27 directly.

### Silencing of *NbGAPDH-A* compromises MP localization to the VRC

To address the possible effects of *NbGAPDH-A* on the subcellular localization of MP, we investigated whether MP targeting to the PD or to the cortical VRC was affected by the silencing of *NbGAPDH-A*. Our previous results showed that the transient expression of MP-GFP in *N. benthamiana* cells resulted in its localization exclusively to the PD, while infection with recombinant RCNMV RNAs that encoded MP-GFP resulted in the formation of cortical VRC and localization to the VRC as well as to the PD [10]. Agroinfiltration of pBICRMsG that expressed MP-GFP fusion protein [10] into ALSV/gsGAP-infected *N. benthamiana* plants resulted in the same localization to PD that was found in ALSV-infected plants (Figure S11). This showed that *NbGAPDH-A* had no effect on the intracellular transportation of RCNMV MP to the PD.

**Figure 9:**
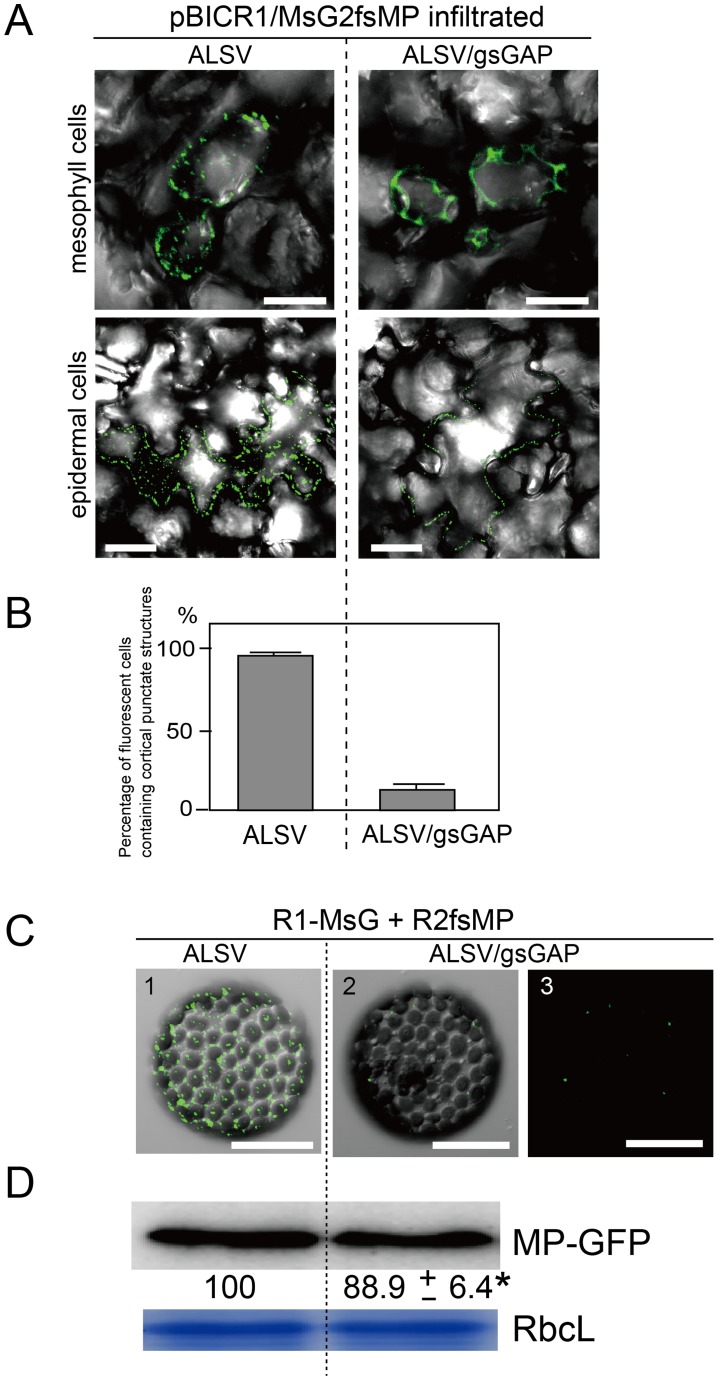
Gene silencing of *NbGAPDH-A* inhibits the localization of RCNMV MP to the cortical VRCs. (**A**) Representative CLSM images of ALSV- or ALSV/gsGAP-infected *N. benthamiana* mesophyll (upper panels) and epidermal cells (lower panels) infiltrated with *Agrobacterium* cultures that contained pBICR1/MsG2fsMP, which expressed recombinant RCNMV RNAs encoding MP-GFP (Figure S1K). The images were obtained at 38 h after infiltration. Scale bar = 30 µm. The images include the merged DIC and GFP channels, and they represent confocal projections of 20 optical sections at 1 µm intervals, ranging from the surface to the middle of the cells. (**B**) The leaf samples in (A) were subjected to epifluorescence microscopy and the percentage of fluorescent cells containing cortical punctates was determined. The data shown are the totals from three replicate assays. (**C**) Protoplasts prepared from ALSV- or ALSV/gsGAP-infected *N. benthamiana* plants were inoculated with a mixture of *in vitro* transcripts of pUCR1-MsG and pRNA2fsMP (Figure S1L) [10]. Representative CLSM images of the protoplasts at 12 hpi. Scale bar = 30 µm. The images include the merged DIC and GFP channels (panels 1 and 2), or GFP channel (panel 3). They represent confocal projections of 15 optical sections at 2 µm intervals, ranging from the surface to the center of the cells. (**D**) The protoplast samples in (C) were subjected to Western blotting. Proteins extracted from 2×10^4^ protoplasts were loaded in each lane. MP-GFP was detected using a rabbit polyclonal antibody against GFP. The numbers below the image represent the relative accumulation levels (means ± SE) of the proteins obtained using the Image Gauge program (Fuji Film), which were calculated from two independent experiments. Asterisk indicates a not significant (P>0.05; Student's *t*-test) difference compared with the accumulation of proteins in the protoplasts from ALSV-infected *N. benthamiana*. RbcL is a Coomassie brilliant blue-stained gel image, which shows the large subunit of Rubisco proteins.

Next, we investigated the effects of *NbGAPDH-A*-silencing on the localization of MP to the cortical VRC. The pBICR1/MsG2fsMP plasmid, which expressed recombinant RCNMV RNAs that encoded MP-GFP (Figure S1K) [10], was agroinfiltrated into ALSV- or ALSV/gsGAP-infected plants. During the early stage of infection at 38 h post infiltration, cortical fluorescent punctates were detected in most of fluorescent mesophyll and epidermal cells in ALSV-infected plants (Figure 9A, left planels), whereas the majority of the fluorescence exhibited a dispersed cytoplasmic pattern in mesophyll cells of ALSV/gsGAP-infected plants (Figure 9A, upper right panels). In epidermal cells, cortical punctates were detected rarely and the PD localization of MP-GFP was detected in ALSV/gsGAP-infected plants (Figure 9A, lower right panel). The ratio of fluorescent cells with cortical punctates was 7.4 times higher in ALSV-infected plants compared with ALSV/gsGAP-infected plants (Figure 9B). At 44 h post infiltration with the recombinant RCNMV, the ratio of fluorescent cells with cortical punctates increased to 41.0% in ALSV/gsGAP-infected plants, although the number of cortical punctates in a single fluorescent cell was lower compared to that in ALSV-infected plants (Figure S12).

The negative effect of *NbGAPDH-A*-silencing on the localization of MP to the cortical VRC was confirmed using protoplasts. At 12 h post infection with the transcripts of pUCR1-MsG and pRNA2fsMP (Figure S1L) [10], cortical fluorescent punctates with MP-GFP were detected in the protoplasts prepared from ALSV-infected plants, whereas they were barely detectable in the protoplasts prepared from ALSV/gsGAP-infected plants (Figure 9C). Probably MP-GFP molecules that were diffused in the cytoplasm could not be detected by CLSM. Despite the reduced fluorescence, MP-GFP accumulated at similar levels in both protoplasts (Figure 9D), thereby showing that *NbGAPDH-A* silencing did not affect the expression, or stability of MP-GFP. These results suggest that NbGAPDH-A is involved in the recruitment of RCNMV MP to the cortical VRC, or that it may stabilize the interaction between MP and VRC.

**Figure 10:**
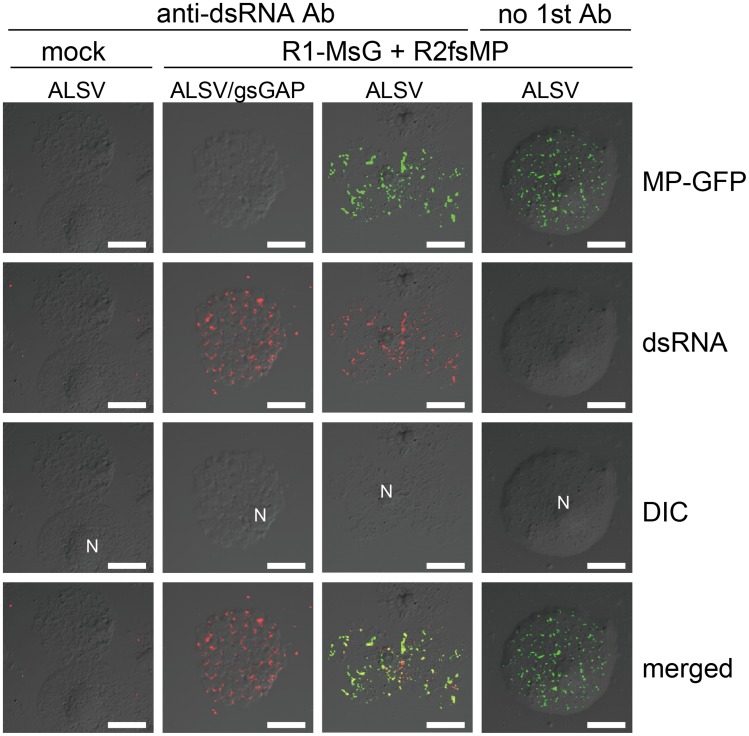
Cortical VRC formation in the *NbGAPDH-A*-silenced *N. benthamiana* protoplasts. Protoplasts prepared from ALSV- or ALSV/gsGAP-infected *N. benthamiana* plants were inoculated with recombinant RCNMV RNAs that expressed the MP-GFP fusion protein (Figure S1L) and subjected to immunostaining with anti-dsRNA primary antibody followed by Alexa Fluor 594-conjugated secondary antibody at an early stage of infection (16 hpi). The left-most panels show the results for mock-inoculated protoplasts treated with the same antibodies. The right-most panels show the results for protoplasts prepared from ALSV-infected plants treated with only secondary antibody. Images present confocal projections of five optical sections at 1 µm intervals, which range from the surface to the middle of the protoplasts. N: nucleus. Scale bar = 20 µm.

Finally, these protoplasts were subjected to immunofluorescent staining of dsRNA for the detection of VRC. In the protoplasts prepared from ALSV/gsGAP-infected plants, MP-GFP was rarely detected. However, cortical punctate-like structures of dsRNA were detected in these cells, as well as in the protoplasts prepared from ALSV-infected plants (Figure 10). The accumulation level of p27 protein was also similar in both protoplasts (Figure S13). These results suggest that NbGAPDH-A does not affect the formation of cortical VRC and that it is associated with the recruitment of RCNMV MP to the cortical VRC.

## Discussion

Replication and movement processes are assumed to be linked to facilitate successful infection by plant viruses. In addition to the temporal regulation of MP expression [47]–[49], spatial regulation is required to allow MPs to encounter the viral genomes. Thus, localized MP synthesis at the VRC, or specific MP recruitment to the VRC, would facilitate efficient and specific virus cell-to-cell movement [50]. In the present study, we showed that MP-containing cortical punctate structures formed during the early stage of RCNMV infection and large aggregates assembled adjacent to the nucleus during the late stage of infection in *N. benthamiana* cells, and both were sites of viral RNA replication (Figure 1). These results suggest that RCNMV VRC changes its location from cortical to perinuclear ER-containing structures, while the VRC also increases in size, as the infection stage proceeds.

A division of roles between the VRCs formed during the early and late stages of infection has recently been proposed for *Potato virus X* (PVX). The early VRCs of PVX are formed in a membranous structure called the ‘cap’ at the orifice of PD, and triple gene block (TGB)-type MPs that accumulate at the cap and PD pore play roles in trafficking the replicated viral genomic RNA via the PD [51]. Furthermore, the X-body formed during the late stage of infection compartmentalizes the TGB1 protein and prevents it from having roles in translational activation, which could lead to the destabilization of PVX virions, while the VRCs that surround the TGB1 core maximize the replication of the viral RNA and the production of virions [52]. The cap structure at the PD orifice and MP compartmentalization in the X-body were not detected in RCNMV-infected cells [10], but it is likely that the two types of RCNMV VRCs have distinct roles. Given that RCNMV cell-to-cell movement occurs before the large aggregate-type VRCs form in virus-infected *N. benthamiana* epidermal cells [10], [39, unpublished results], it is probable that only the cortical VRC contributes to virus cell-to-cell movement whereas the large aggregate-type VRC (X-body) might maximize the production of progeny virions.

Our previous studies showed that the host proteins that contribute to the replication of RCNMV RNAs colocalized with p27 in the perinuclear large aggregates [30], [31], rather than the cortical VRC. It is possible that the modes of VRC formation differ between the cortical VRC and the perinuclear large aggregates. Further studies using specific antibodies against the host factors associated with the VRC are required to answer this question.

We identified NbGAPDH-A as an interacting partner for RCNMV MP (Figure 2). Although VIGS of *NbGAPDH-A* using ALSV vector that contained 294 bases of the gene fragment reduced the accumulation of the mRNA to 3% of that in the empty ALSV infected plants (Figure 3A), the silencing had no effect on plant growth (Figure S4). This result contradicted a previous report where transgenic tobacco (*Nicotiana tabacum*) plants with silenced *GAPDH-A* exhibited severe growth inhibition compared with the wild type plants [53]. Over 1,000 bases of the *GAPDH-A* coding region had been introduced into these transgenic plants to express an antisense RNA that was complementary to *GAPDH-A* mRNA. The induction of gene silencing using such a long sequence might have affected the expression levels of unidentified GAPDH orthologs, which could have led to growth inhibition. Alternatively, *N. tabacum* GAPDH-A might have a greater impact on growth than that of *N. benthamiana*, or transgenic plants in which the gene was silenced had a greater effect on the phenotype than VIGS. Our preliminary results showed that induction of VIGS of *NbGAPDH-A* by the other widely-used VIGS vector based on *Tobacco rattle virus* (TRV) caused the same symptoms as those by the empty TRV vector (Figure S14). This supports that VIGS of *NbGAPDH-A* in *N. benthamiana* does not cause severe symptoms.

We showed that NbGAPDH-A is a host protein that is involved in the cell-to-cell movement of RCNMV (Figures 3 and 4). In addition to chloroplast localization, the NbGAPDH-A-GFP fusion protein also localized to cortical VRCs. The localization to VRC was associated with viral RNA replication, not the replicase component proteins alone (Figure 5B). Gene silencing of *NbGAPDH-A* inhibited the targeting of RCNMV MP to cortical VRCs (Figure 9), but it did not affect the targeting of MP-GFP to the PD (Figure S11), or the stability of MP (Figures 4 and 9). Based on the overall results obtained in the present study, we propose that NbGAPDH-A is contained in the VRC without influencing viral RNA replication and is an interstitial agent between RCNMV MP and the VRC. NbGAPDH-A probably plays a role in recruiting MP to the VRC, or stabilizing the interaction between MP and VRC. BiFC assays confirmed the *in vivo* interaction between NbGAPDH-A and the MP (Figure 7). BiFC assays also showed that the interaction occurred not only in the cortical VRC, but also in the large aggregates and in the cell wall (Figure 7B). The distribution pattern of the reconstituted YFP signal was quite similar to that of the MP-GFP expressed from the recombinant virus [10]. The signals of the reconstituted YFP observed in the cell wall could be due to the NbGAPDH-A-YFP-MP complexes that had been transported to PD by the function of MP. However, significance of the observed colocalization of NbGAPDH-A and MP in the cell wall is ambiguous. This is because the reconstitution of YFP is irreversible [54], and because NbGAPDH-A-GFP did not localize in the cell wall when coexpressed with RCNMV RNA1 and RNA2 (Figure S7). Further study is needed to elucidate the role, if any, of NbGAPDH-A in the cell wall in virus infection.

GST pulldown assays confirmed that NbGAPDH-A interacted with both p27 and MP *in vitro* (Figure 8). This suggests that NbGAPDH-A may be a bridge between MP and p27 that is a constituent of the VRC. *In vivo*, however, relocalization of NbGAPDH-A-GFP to the cortical VRC and interaction of NbGAPDH-A-cYFP and MP-nYFP occurred only in association with the viral RNA replication (Figures 5B and 7). By contrast, coexpression of NbGAPDH-A-GFP and p27 fused with DsRed-monomer in *N. benthamiana* cells using agroinfiltration resulted in different localization patterns, with the former in chloroplasts and the latter in the ER-containing large aggregate ([32] and Figure S15). Furthermore, coexpression with MP-mCherry did not affect the localization of NbGAPDH-A-GFP to the chloroplasts (Figure 5A). These results suggest that unidentified factors might be involved in the *in vivo* interaction between NbGAPDH-A and viral proteins. Three possibilities can be considered, 1) Enhancement of the local concentration of viral proteins: RNA1 replicates autonomously, and the replication coupled with the translation of replicase proteins p27 and p88, followed by the formation of 480 kDa replication complex [30], [55], [56]. This replication cycle might increase the local concentration of p27 in or near the VRC on the cortical ER membrane to higher levels than transiently expressed p27. Such a process might improve the probability that p27 and NbGAPDH-A will encounter in the cortical VRC. In association with this assumption, transiently expressed p27-GFP alone forms a large aggregate ([32] and Figure S15). Formation of such aggregates might sequester p27 and prevent the interaction with NbGAPDH-A. 2) Involvement of unknown host proteins associated with the VRC: The formation of the 480 kDa replication complex of RCNMV requires not only p27 and p88 but also viral RNAs in host cells [30]. The 480 kDa replication complex contains many host proteins that have not been identified yet. It is possible that such unknown proteins are involved in the recruitment of NbGAPDH-A to the VRC or in the stabilization of the interaction *in vivo*. 3) Involvement of viral RNA: Cytoplasmic GAPDH (GAPDH-C) has been reported to interact with the *cis*-acting elements of many RNA viruses, some of which affect the multiplication of viruses ([57], [58] and references therein). Although chloroplastic and cytoplasmic GAPDHs are assumed to have evolved from different lineages [59], their amino acid sequence identity is as high as ca 45% in *Arabidopsis thaliana* (NCBI Gene ID: 819567 and 822277) and *Zea mays* (NCBI Gene ID: 542367 and 542368). It is possible that NbGAPDH-A also has an RNA-binding ability and that it is recruited to VRCs by interacting with RCNMV RNAs *in vivo*, as shown in *Hepatitis delta virus*-infected cultured cells [60]. These three possibilities may not be mutually exclusive. Further studies on the molecular mechanisms of the VRC formation are awaited.

In addition to glycolysis, cytoplasmic GAPDH-C has a variety of functions, such as membrane fusion and vesicular transport (reviewed in [61], [62]). In contrast, only the classical functions associated with carbon fixation have been reported previously for GAPDH-A. Thus, further experimental evidence is required to explain the involvement of GAPDH-A in the intracellular transport mechanism of RCNMV RNA. Alternatively, RCNMV might have evolved to utilize highly expressed and ubiquitous GAPDH-A by diverting it from its natural functions. Several plant RNA viruses use host metabolic enzymes and housekeeping proteins in ways that are unrelated to their original functions [63]. Several chloroplast-localizing proteins have recently been shown to regulate virus multiplication. Among these, chloroplastic phosphoglycerate kinase (PGK) was isolated from RNA-dependent RNA polymerase (RdRp) fraction prepared from *Bamboo mosaic virus*-infected *N. benthamiana*. PGK positively regulates multiplication of the virus through the interaction with the 3′ untranslated region of the viral genomic RNA and transportation to the chloroplasts where the viral RNA replication occurs [64], [65]. ATP synthase-γ subunit (AtpC) and Rubisco activase (RCA) were also isolated from the RdRp fraction prepared from the TMV-infected *N. tabacum*. AtpC and RCA negatively regulate the movement and accumulation of the virus, respectively [66]. Interestingly, gene silencing of these genes led to the increased number and the smaller size of VRC. These results are in contrast to our results that VIGS of *NbGAPDH-A* did not affect the number and the size of the cortical VRC in RCNMV infected cells and that it interfered with the recruitment of the MP to the cortical VRC (Figures 9 and 10). Plant RNA viruses might have evolved to utilize abundant chloroplast-localizing proteins as the positive or negative regulators through the interaction with viral proteins.

## Materials and Methods

Plasmids given the prefix “pBIC” were used for *Agrobacterium* infiltration, “pUC”, “pRC” and “pR” were used for *in vitro* transcription, “pCold” was used for protein expression in *Escherichia coli*. pUCR1 [67] and pRC2|G [68] are full-length cDNA clones of RNA1 and RNA2 of an RCNMV Australian strain, respectively (Figure S1A). The plasmids described previously used in this study include pUCR1-MsG (Figure S1L) [39], pBICR12fsMP (Figure S1G) [10], pBICRMsG [10], pBICR1/MsG2fsMP (Figure S1K) [10], pRNA2fsMP (Figure S1B) [10], pBICP35 [39], pBICp27-iFTH [28], pColdGST (where GST is glutathione *S*-transferase) [29], pColdGSTp27 [29], pBICHA:cYFP [30], pBICp27 [67], pBICp88 [67], and pBICp19 [67].

*E. coli* DH5α was used for the construction of all plasmids. All PCR reactions were performed using a high fidelity proofreading KOD Plus-Ver.2 polymerase (Toyobo), and all the PCR-amplified regions were verified by sequencing. The primers used in this study are listed in Table S2.

### Construction of plasmids expressing recombinant virus RNAs

mCherry gene was amplified from pmCherry-N1 (Clontech) using primers 1 and 2. The amplified PCR product was digested with *Cla*I/*Mlu*I and inserted into the same sites of pUCR1-MsG [39], producing pUCR1-MmC (Figure S1B) that expresses MP-mCherry fusion protein from the subgenomic RNA.

*Asc*I/*Sac*I fragment of pUC118RA1(AscI) [10] that contains the expression cassette of RCNMV RNA1 and *Asc*I/*Sma*I fragment of pUC118RA2(AscI) [10] that contains the expression cassette of RCNMV RNA2 was inserted into *Sac*I/*Sma*I site of pBIC18 [67] binary vector, producing pBICR12 (Figure S1C) that expresses full-genome of RCNMV.

pBICp27-iFTH is a binary vector plasmid expressing p27 tagged with FLAG-TEV protease recognition peptide-HA [28]. The TEV protease recognition peptide was replaced by 3C protease recognition peptide by recombinant PCR to produce pBICp27TEP (Mine and Okuno, unpublished). The tag sequence for tandem affinity purification was amplified from pBICp27TEP using primers 3 and 4. The cauliflower mosaic virus 35S promoter and 5′ half of RNA2 was amplified from pBICR12 using primers 5 and 6. A DNA fragment containing the 3′ half of RNA2 and the 35S terminator sequence was amplified from pBICR12 using primers 7 and 8. These three fragments were mixed and used as the template for recombinant PCR using primers 6 and 8. The generated PCR product was digested with *Asc*I/*Sma*I and inserted into the same sites of pBICR12, producing pBICR12/MP-TAP (Figure S1D).

R1-MP:GFP plasmid [38] was digested with *Cla*I and MP coding sequence was removed. The larger fragment was self-ligated, producing pR1-sGFP (Figure S1E).

pR1-sGFP was digested with *Bgl*II/*Mlu*I and the 0.8 kb fragment containing GFP gene was inserted into the same sites of pUC118RA1(AscI), producing pUC118RA1sGC(AscI). pUC118RA1sGC(AscI) was digested with *Asc*I/*Sac*I and the 4.4 kb fragment was inserted to the same sites of pBICR12, producing pBICR1sG2 (Figure S1F).

A DNA fragment containing HA and the 3′ non-coding region of RNA2 was amplified from pRC|2G using primers 9 and 10. A DNA fragment containing T7 promoter and the 5′ half of RNA2 and HA was amplified from pRC2|G using primers 11 and 12. These fragments were mixed and used as the template for recombinant PCR using primers 10 and 12. The generated PCR product was digested with *Eco*RI/*Sma*I and inserted into the same site of pUC119, producing pUCR2MP-HA (Figure S1H).

*Eco*RI/*Hin*dIII fragment of pBE2113 [69] that contains a 35S promoter-∧ sequence-nos terminator cassette was inserted to the same sites of pUC19 (Takara Bio Inc.) producing pUC2113. The *Xba*I site downstream of ∧ sequence in pUC2113 was digested and filled in with T4-polymerase, and the linker sequence containing *Sac*I site was ligated, producing pUC2113(*Sac*I). *Eco*RV fragment of piL:G3 (0.4 kb) [70] containing the 35S promoter and the 5′ sequence of *Tomato mosaic virus* (ToMV) was inserted to the same site of pUC2113(*Sac*I), producing pUC:ToMVrec. *Eco*RI/*Hin*dIII fragment of pBE2113 was replaced by the *Eco*RI/*Hin*dIII fragment of pUC:ToMVrec, producing pBE:ToMVrec. The *Kpn*I/*Mlu*I fragment of piL:G3 containing ToMV MP and GFP sequence was inserted to the same sites of pTLW3 [71], producing pTLWdCP-GFP. The ribozyme sequence of *Tobacco ringspot virus* satellite RNA was PCR-amplified from pUCBR1R plasmid [72] and introduced to *Mlu*I site downstream of ToMV 3′ noncoding sequence in pTLWdCP-GFP, producing pTLWdCP-GFP-rib. Finally, *Stu*I/*Sac*I fragment of pTLWdCP-GFP-rib, containing most of the recombinant ToMV and the ribozyme sequences was inserted into the same sites of pBE:ToMVrec, producing pToMVdCP-GFP.

### Plant growth conditions

*N. benthamiana* plants were grown on commercial soil (Tsuchi-Taro, Sumirin-Nosan-Kogyo Co. Ltd.) at 25±2°C and 16 hours illumination per day.

### Protoplast preparation and viral RNA inoculation using polyethylene glycol

*N. benthamiana* protoplasts were prepared according to Li *et al.* (2013) [73] and Navas-Castillo *et al.* (1997) [74] with minor modifications. Briefly, young expanded leaves from 5 week-old plants were cut into 1-mm strips with a razor blade and digested in 15 ml of enzyme solution (1% cellulase RS [Yakult Pharmaceutical Ind. Co. Ltd.], 0.5% macerozyme R-10 [Yakult], 0.5 M mannitol, 10 mM CaCl_2_, 5 mM MES, pH 5.7) within a petri dish at 25°C in the dark with gentle shaking (40 rpm) for 4 to 5 h. After being filtered through 4 layers of cheesecloth, protoplasts were precipitated by centrifugation at 80× g for 2 min and were suspended with 10 ml of MMC solution (0.5 M mannitol, 10 mM CaCl_2_, 5 mM MES, pH 5.7). Concentration of cells were counted using hemacytometer. Protoplasts (1×10^5^ cells in 100 µl) were mixed with 5 µg of viral RNAs and 200 µl of PEG solution (1 g of PEG4000 [Sigma-Aldrich #81240], 125 µl of sterile distilled water, 1.25 ml of 0.8 M mannitol, 250 µl of 1 M Ca(NO_3_)_2_) and mixed completely by gently tapping the tube. Then 2 ml of MMC solution was added and mixed. After 15 min of incubation on ice, protoplasts were precipitated by centrifugation at 80× g for 2 min, resuspended in 4 ml of MMC solution and precipitated by centrifugation again. Protoplasts were resuspended in 0.5 ml of W5 solution (154 mM NaCl, 125 mM CaCl_2_, 5 mM KCl, 2 mM MES, pH 5.7) and incubated.

Preparation of protoplasts from *NbGAPDH-A*-silenced or ALSV-infected *N benthamiana* plants was essentially described above, except that the plants were 6–8 weeks old.

### Immunofluorescent labeling

Fixation of *N. benthamiana* protoplasts and immunolabeling procedure were as described by Liu *et al.* (2005) [20]. For the detection of double-stranded RNA, formaldehyde-fixed protoplasts were incubated with mouse monoclonal antibody J2 (diluted 1∶200; Scicons) for 16 h in a moisturized chamber at 4°C. The samples were washed three times and then incubated with Alexa Fluor 488-conjugated goat anti-mouse IgG antibody (diluted 1∶200; Invitrogen) for 2 h at room temperature. After washing three times, the samples were subjected to CLSM.

### Affinity purification of RCNMV MP-containing fraction

*N. benthamiana* plants and *Agrobacterium tumefaciens* GV3101 (pMP90) were used for infiltration experiments as described previously [67]. *A. tumefaciens* transformed by pBICR12/MP-TAP, or negative control pBICR12 was used for expression of MP-HA from viral context. 1.67 g of *Agrobacterium*-infiltrated leaves at 48 h post infiltration were ground in liquid nitrogen and homogenized in 5 ml of extraction buffer A (50 mM Tris-HCl [pH 8.0], 150 mM NaCl, 5% glycerol, 0.5% Triton X-100, 1 tablet of Complete Mini protease inhibitor cocktail [EDTA-free, Roche Diagnostics]/10 ml), followed by centrifugation at 21,000× g for 10 min at 4°C to remove cell debris. The supernatant (4.0 ml) was divided into 5 tubes (800 µl each), and each incubated with 20 µl of Anti-HA Affinity Matrix (Roche #11815016001) for 4 h at 4°C with gentle rotation. The resin was washed three times with 1 ml of washing buffer 1 (50 mM Tris-HCl [pH 7.4], 150 mM NaCl, 5% glycerol, 0.1% Triton X-100), and equilibrated with 3C buffer (washing buffer 1 containing 1 mM DTT). Then the resin was incubated with 20 units of PreScission protease (GE Healthcare) in 1 ml of 3C buffer for 16 h at 4°C with gentle rotation. The resins were centrifuged at 500× g for 1 min, and the supernatant (1 ml) was immunoprecipitated again with 50 µl of ANTI-FLAG M2 Affinity Gel (Sigma-Aldrich #A2220) for 4 h at 4°C with gentle rotation. The gel was then washed three times with washing buffer 1. The bound proteins were eluted by 125 µl of elution solution (washing buffer 1 containing 150 ng/µl FLAG peptide [Sigma-Aldrich #F3290]) for 30 min at 4°C with gentle rotation. This elution process was repeated once again, and the total of 250 µl was precipitated with trichloroacetic acid.

The affinity-purified preparation and its control preparation were subjected to SDS-PAGE, and the several bands that were not detected in the negative control lane (Figure 2A) were cut out and subjected to liquid chromatography-tandem mass spectrometry analysis, as described previously [28].

### Cloning of *NbGAPDH* genes

RNA extraction from *N. benthamiana* leaves was performed using PureLink Plant RNA Reagent (Invitrogen) and treated with DNase (RQ1 RNase-free DNase; Promega). Reverse transcription was carried out using PrimeScript RT reagent Kit (Takara) using oligo-dT.

Based on the *GAPDH-A* sequence of *N. tabacum* (gi|120661), primers 13 and 14 were designed. An 1176-bp nearly full-length cDNA fragment of *GAPDH-A* gene was amplified from cDNA derived from *N. benthamiana* RNA using primers 13 and 14. The 5′ and 3′ sequences of *NbGAPDH-A* were amplified by SMARTer RACE cDNA amplification Kit (Clontech) using the gene-specific primers 15 and 16, respectively, and cloned into pGEM-T Easy (Promega). From each of 8 clones the 5′ and 3′ ends of *NbGAPDH-A* gene were determined. Nucleotide sequence data of *NbGAPDH-A* gene is available in the DDBJ/EMBL/Genebank databases under accession number AB937979.

Based on the *GAPDH-B* sequence of *N. tabacum* (gi|120665), primers 17 and 18 were designed. A 1270-bp partial fragment of *GAPDH-B* cDNA was amplified from cDNA derived from *N. benthamiana* RNA using primers 17 and 18, and cloned into pGEM-T Easy. Partial sequence of *NbGAPDH-B* gene was determined.

### Construction of plasmids that express NbGAPDH-A-derivatives

Full-length cDNA of *NbGAPDH-A* was amplified from cDNA derived from *N. benthamiana* RNA using primers 19 and 20. The generated PCR product was then cloned into the *Bam*HI site of pBICP35, producing pBICNbGA-myc.

Full-length cDNA of *NbGAPDH-A* was amplified from pBICNbGA-myc using primers 14 and 19. The generated PCR product was digested with *Bam*HI/*Cla*I and cloned into the same sites of pUB/RMsG [39], producing pUBNbGA-sG. *Bam*HI/*Hin*dIII fragment of pUBNbGA-sG, containing *NbGAPDH-A-GFP* and 35S terminator, was cloned into the same sites of pBICP35, producing pBICNbGA-sG. This was used for transient expression of NbGAPDH-A-GFP fusion protein by agroinfiltration.

### Construction of plasmids that express fluorescent protein-tagged markers

Chloroplast targeting sequence of RbcS was amplified from cDNA derived from *A. thaliana* RNA using primers 21 and 22. sGFP sequence was amplified from pUBsGFP [39] using primers 23 and 24. Recombinant PCR fragment was amplified using primers 21 and 24. The generated PCR product was then cloned into the *Bam*HI and *Kpn*I sites of pUBP35 [67], producing pUBTPRbcS-sGFP. *Hin*dIII/*Sal*I fragment of pUBTPRbcS-sGFP was cloned into the same sites of pBICP35, producing pBICRbcSTP-sGFP.

MP-mCherry sequence was amplified from pUCR1-MmC using primers 25 and 26. The generated PCR product was digested with *Bam*HI/*Eco*RI and cloned into the same sites of pBICP35, producing pBICRMmC.

pBICRbcSTP-sGFP and pBICRMmC was introduced into *A. tumefaciens* and used for the transient expression of RbcSTP-GFP and MP-mCherry, respectively.

### Virus-induced gene silencing

Construction of ALSV vector pBICAL1, pBICAL2 and pBICAL2gsPDS was described previously [75]. Partial fragment of *NbGAPDH-A* (294 nucleotides) was amplified from cDNA derived from *N. benthamiana* RNA using primers 27 and 28. The fragment was digested with *Bam*HI/*Xho*I and inserted into the same sites of pBICAL2, producing pBICAL2gsNbGAP-A.

The plasmids containing the ALSV expression cassette were introduced into *A. tumefaciens* GV3101 (pMP90). Similar amount of fresh colonies of *Agrobacterium* containing pBICAL1 and each of pBICAL2, pBICAL2gsPDS, or pBICAL2gsNbGAP-A were collected using sterile toothpicks and suspended in 0.2 ml of Agro Incubation Buffer (10 mM MgCl_2_, 10 mM MES-KOH, pH 5.7, 0.15 mM Acetosyringone) at OD_600_ of 2.0–3.0, and were incubated at 20°C for more than 3 h in the dark. Sterile toothpick was soaked in the *Agrobacterium* suspension and stuck 4 times into 1st, 2nd and 3rd true leaves of 17–21 days old *N. benthamiana* plants. Three to 4 days later, toothpick-inoculation was repeated to a newly developed leaf. After inoculation, the plants were incubated in a moist chamber at 22°C overnight and transferred to a plant growth room at 25°C. Two to 3 weeks later, silencing of *PDS* or *NbGAPDH-A* was induced in the non-inoculated upper leaves.

### Quantitative and semi-quantitative RT-PCR analysis

Total RNA extracted from *N. benthamina* leaves or protoplasts were subjected to reverse transcription using PrimeScript RT reagent Kit (Takara) using oligo-dT according to manufacturer's protocol. Real-time PCR was carried out using SYBR Premix Ex Taq (RR420A, Takara) using primers 29 and 30 for EF1 and primers 16 and 31 for *NbGAPDH-A*. Quantitative analysis of each mRNA was performed using a Thermal cycler Dice Real Time System TP800 (Takara). Semi-quantitative RT-PCR was performed using the same cDNA and primers and amplified by Ex Taq polymerase (Takara).

### Western and northern blot analyses

Protein extraction and western blot analyses were performed as described previously [76]. Total RNA extraction from *N. benthamiana* leaves or protoplasts and northern blot analysis were performed as described previously [76]. Probes used for detection of positive-strand RCNMV RNA1 and RNA2 were as described previously [49]. The signals were detected with a luminescent image analyzer (LAS 1000 plus, Fuji Film Co. Ltd.) and the signal intensities were quantified using the Image Gauge program version 3.1 (Fuji Film).

### Microscopy

The spread of GFP fluorescence was observed using an Olympus BX53 fluorescence microscope equipped with an Olympus DP72 camera using the imaging program Olympus cellSens.

Subcellular localizations of proteins tagged with FPs and dsRNA that was detected with fluorescent antibodies were observed using an Olympus FluoView FV500 confocal microscope. Both a Nikon 60× Plan Apo oil immersion objective lens (numerical aperture 1.4) and a Nikon 40× UPlan Apo oil immersion objective lens (numerical aperture 1.0) were used. The sets of dichroic mirror, beam splitter, and emission filter used were DM488/543, SDM560, and BA505-525 for GFP, and DM488/543/633, SDM630, and BA560-600 for mCherry For the detection of mCherry signal and chloroplast autofluorescence simultaneously, emission filter BA610IF was used. In experiments for detecting dual localization, scanning was performed in sequential mode to minimize signal bleed-through. All images shown are from optical sections taken at 1 or 2 µm intervals and were processed using Adobe Photoshop CS6 software.

### Construction of protein-expression vectors in *E. coli*

*Bam*HI/*Eco*RI fragment of pBICRMP-HA [39] that contains MP-HA gene was inserted into pCold-1 (Takara), producing pCold-MP-HA. *Eco*RI/*Kpn*I fragment of pCold-MP-HA that contains MP-HA gene was inserted into the same sites of pColdGST, producing pColdGST/MP-HA.

pBICNbGAP-myc was digested with *Bam*HI and the smaller fragment containing *NbGAPDH-A-myc* was cloned into the same site of pCold-1 in the correct orientation, producing pColdNbGAP-myc.

### GST pulldown assay

*E. coli* BL21(DE3) strain was transformed with plasmids containing the prefix pCold and used for the expression of GST and GST-fused viral proteins and NbGAPDH-A tagged with a myc. All the conditions and procedures are described previously [31].

### Construction of BiFC vectors

The sequence of the C-terminal half of YFP was amplified from pBICHA:cYFP [30] using primers 32 and 33 [30]. The amplified DNA was digested with *Stu*I and cloned into *Stu*I-digested pBICAsc2 [30], producing pBICHA-cYFPAsc2. Full-length cDNA of *NbGAPDH-A* was amplified from pUBNbGA-sG using primers 34 and 35. The generated PCR product was digested with *Bam*HI and cloned into pBICHA-cYFPAsc2, producing pBICGAP-HA-cYFP.

myc-nYFP sequence was amplified from pBICMP-myc-nYFP using primers 36 and 37. The amplified PCR products were digested with *Cla*I/*Mlu*I and cloned into the same sites of pUCR1-MsG, producing pUCR1-MnY (Figure S1I).

p88-myc-nYFP sequence was amplified from pUCR1-MnY using primers 37 and 38. p88 sequence was amplified using primers 39 and 40 The recombinant PCR products were generated from mixture of these products using primers 37 and 39, and were digested with *Mlu*I/*Xho*I and cloned into the same sites of pUCR1-MsG, producing pUCR1-nYFP (Figure S1J).

### BiFC assay

Twenty-five to 28 days old *N. benthamiana* plants were used for BiFC assays. pBICGAP-HA-cYFP, or control pBICHA-cYFPAsc2 plasmid, together with pBICp19 that expresses TBSV silencing suppressor protein p19 was infiltrated via *A. tumefaciens* GV3101 (pMP90) as described above. The plants were incubated in a moist chamber at 22°C for 18 h. Then *in vitro* transcripts (1 µg/µl) of the recombinant RCNMV that expresses MP-nYFP (Figure S1I), or negative control virus that expresses nYFP (Figure S1J) were mechanically inoculated onto the leaves. The plants were incubated in a moist chamber at 17°C for 27–30 h and were subjected to CLSM.

### Accession number

*NbGAPDH-A* was registered through DDBJ and accession number AB937979 was given on May 27 2014.

## Supporting Information

Figure S1 **Schematic diagrams of** ***Red clover necrotic mosaic virus*** **(RCNMV) and various derivative constructs.** (**A**) Genome map of RCNMV. Open boxes and bold lines show open reading frame (ORF) and the untranslated regions of the virus, respectively. (**B–L**) Plasmids containing the prefix ‘pUC’ and ‘pR’ and pRNA2fsMP were cut with *Sma*I and used as templates for *in vitro* transcription. Plasmids containing the prefix ‘pBIC’ were used for inoculation via *Agrobacterium*. Shaded boxes show the ORF of fluorescent proteins and tag peptides. Dashed boxes show the untranslated MP ORF; and fs is the four-nucleotide insertion for a frameshifting mutation. Key: T7, T7 promoter; Pro, *Cauliflower mosaic virus* (CaMV) 35S promoter; Ter, CaMV terminator; Rz, ribozyme sequence; SmaI, *Sma*I recognition sequence.
(TIF)

Figure S2 **RCNMV MP fused with tandem affinity purification (TAP) tag sequence is functional.** pBICR12 (Figure S1C) and pBICR12/MP-TAP (Figure S1D) and pBICR12fsMP (Figure S1G) [10] were inoculated to two young *N. bethamiana* plants via *Agrobacterium* using toothpicks (see ‘Virus-induced gene silencing’ paragraphs in Materials and Methods), respectively. Proteins were extracted from the inoculated leaves at 4 days post infiltration (dpi) and upper non-inoculated leaves at 7 dpi, respectively. 20 µg of samples was loaded to each lane. CP was detected using a rabbit polyclonal antibodies against RCNMV CP. MP-TAP was detected using a rat polyclonal antibodies against HA. RbcL is a Coomassie brilliant blue-stained gel image, which shows the large subunit of Rubisco proteins.
(TIF)

Figure S3 **Semi-quantitative RT-PCR analysis of NbGAPDH-A mRNA accumulation levels in the ALSV vector-infected plants leaves.** Total RNA was prepared from each of two independent plants inoculated with empty ALSV vector or ALSV/gsGAP vector. *NbGAPDH-A* mRNA levels were determined by semi-quantitative RT-PCR. The RT-PCR results for the *EF-1* gene show that equal amounts of total RNA were used for RT, and the RT reaction had an equivalent efficiency with the samples. Primers used to amplify both genes are similar to those used in Figure 3A.
(TIF)

Figure S4 ***NbGAPDH-A*** **-silenced plant as well as ALSV-infected plant does not exhibit any symptoms.** Representative images of *N. benthamiana* plants 26 days post inoculation with ALSV vectors via *Agrobacterium*. *N. benthamiana* plants inoculated with the vector containing 102 nt of *Phytoene desaturase* (ALSVgsPDS) started to be white at 9 dpi. Infection with ALSV empty vector (wt ALSV) and the vector containing 294 nt of *NbGAPDH-A* gene (ALSVgsGAP) did not affect plant growth and no symptoms were detected.
(TIF)

Figure S5 **Multiplication of a recombinant RCNMV in** ***NbGAPDH-A*** **-silenced** ***N. benthamiana*** **leaves at a late stage of infection.** An *Agrobacterium* culture that contained the pBICR1sG2 plasmid, which expressed RCNMV-GFP (Figure S1F), was diluted to OD_600_ = 0.03 and infiltrated into ALSV- or ALSV/gsGAP-infected *N. benthamiana* plants. At 48 hpi, protein was extracted from the infiltrated leaves and subjected to Western blotting using anti-GFP antibody. RbcL is a Coomassie brilliant blue-stained gel image, which shows the large subunit of Rubisco proteins. The accumulated levels of GFP from three separate experiments were quantified using the Image Gauge program and plotted in the graph.
(TIF)

Figure S6 **Multiplication of** ***Tomato mosaic virus*** **is not affected by the silencing of** ***NbGAPDH-A*** **.** An *Agrobacterium* culture that contained the pToMVdCP-GFP plasmid, which expressed the recombinant *Tomato mosaic virus* in which the CP gene was replaced by GFP gene was diluted to OD_600_ = 0.03 and infiltrated into ALSV- or ALSV/gsGAP-infected *N. benthamiana* plants. At 40 and 48 hpi, protein was extracted from the infiltrated leaves and subjected to Western blotting using anti-GFP antibody. RbcL is a Coomassie brilliant blue-stained gel image, which shows the large subunit of Rubisco proteins. The lower panels are the representative epifluorescence microscopy images of the infiltrated leaves at 40 hpi and 48 hpi. Scale bar = 100 µm.
(TIF)

Figure S7 **Subcellular localization of NbGAPDH-A-GFP coexpressed with RCNMV RNA1 and RNA2.** Representative CLSM images of agroinfiltrated *N. benthamiana* epidermal cells which transiently expressed NbGAPDH-A-GFP with both RCNMV RNA1 and RNA2. Arrowheads represent cortical NbGAPDH-A-GFP signals and arrows represent chloroplast-localized NbGAPDH-A-GFP signal. Other conditions for infiltration and CLSM observation are similar to those in Figure 5. Scale bar = 20 µm.
(TIF)

Figure S8 **Accumulation of p27 and NbGAPDH-A in the agroinfiltrated leaves.** NbGAPDH-A was expressed in *N. benthamiana* leaves together with RCNMV RNA1, or RCNMV replicase proteins p27 and p88. Each *Agrobacterium* culture was diluted to OD_600_ = 0.8 and equal volume of cultures were mixed and infiltrated into *N. benthamiana* leaves. At 40 hpi, protein was extracted from the infiltrated leaves and subjected to Western blotting using anti-p27 and anti-GFP antibodies. RbcL is a Coomassie brilliant blue-stained gel image, which shows the large subunit of Rubisco proteins.
(TIF)

Figure S9 **Accumulation of RNA1 in the agroinfiltrated leaves.** An *Agrobacterium* culture that contained the plasmid that expressed RbcSTP-GFP, GFP, RCNMV RNA1 and that contained control vector plasmid was diluted to OD_600_ = 0.8. Equal volume of each combination of cultures was mixed and infiltrated into *N. benthamiana* plants. At 40 hpi, the total RNA was extracted from the infiltrated leaves and subjected to Northern blotting using DIG-labeled riboprobes specific for the plus (+)- and minus (−)-strand RNA1 of RCNMV. *In vitro* transcripts of (+)-RNA1 (10 µg) and (−)-RNA1 (1 µg) were loaded as the control marker. rRNA is an ethidium bromide-stained agarose gel image of 1 µg total RNA, which was used as the loading control.
(TIF)

Figure S10 **RNase treatment does not affect the interaction between NbGAPDH-A and RCNMV proteins** ***in vitro*** **.** In the presence (+) or absence (−) of RNase A (50 µg/ml), glutathione resin-bound proteins were incubated with His-GAP-myc for 2 h at 4°C. The beads were then washed and the pulled-down complexes were subjected to SDS-PAGE and analyzed by Western blotting (Wb) using anti-GST and anti-myc antibodies.
(TIF)

Figure S11 **PD targeting of RCNMV MP-GFP is not affected by the silencing of NbGAPDH-A.** *Agrobacterium* culture containing pBICRMsG plasmid that transiently expresses RCNMV MP-GFP under the control of *Cauliflower Mosaic Virus* 35S promoter [10] was diluted to OD_600_ = 0.8 and infiltrated into ALSV- or ALSV/gsGAP-infected *N. benthamiana* plants. Representative CLSM images of the leaves at 35 hpi show that the MP-GFP localized to the PD irrespective of the silencing of *NbGAPDH-A*. Scale bars = 20 µm. Images present confocal projections composed of 5 optical sections taken at 1 µm intervals, around cell wall region (top 2 panels) or cortical surface region (lower 4 panels) of epidermal cells.
(TIF)

Figure S12 **Localization of RCNMV MP-GFP in** ***NbGAPDH-A*** **–silenced plants at a later stage of infection of the recombinant virus that expresses MP-GFP.** Representative CLSM images of ALSV/gsGAP-infected *N. benthamiana* mesophyll cells infiltrated with *Agrobacterium* cultures that contained pBICR1/MsG2fsMP, which expressed recombinant RCNMV RNAs encoding MP-GFP (Figure S1K). The images were obtained at 44 h after infiltration. Scale bar = 30 µm. The images represent confocal projections of 20 optical sections at 1 µm intervals, ranging from the surface to the middle of the cells.
(TIF)

Figure S13 **Accumulation of p27 in the protoplasts prepared from ALSV or ALSV/gsGAP-infected plants.** Protoplasts prepared from ALSV- or ALSV/gsGAP-infected *N. benthamiana* plants were inoculated with recombinant RCNMV RNAs that expressed the MP-GFP fusion protein (Figure S1L). Protein was extracted Proteins extracted at 16 hpi from 2×10^4^ protoplasts were loaded in each lane. p27 was detected using the protein-specific rabbit polyclonal antibody. RbcL is a Coomassie brilliant blue-stained gel image of proteins extracted from 2×10^4^ protoplasts, which shows the large subunit of Rubisco proteins. The left-most panels show the results for mock-inoculated protoplasts treated with the same antibodies.
(TIF)

Figure S14 ***NbGAPDH-A*** **-silenced plant by TRV-based vector exhibits similar mild symptoms as that by empty TRV vector-infected plant.** Representative images of *N. benthamiana* plants 25 days post inoculation with TRV vectors via *Agrobacterium*. Infection with TRV empty vector (wt TRV) and the vector containing 294 nt of *NbGAPDH-A* gene (TRVgsGAP) did not affect plant growth and similar mild symptoms were detected.
(TIF)

Figure S15 **Subcellular localization of NbGAPDH-A-GFP is not affected by the coexpression of p27-DRm.** Representative CLSM images of agroinfiltrated *N. benthamiana* cells. Each *Agrobacterium* culture was diluted to OD_600_ = 0.8 and equal volume of cultures were mixed for infiltration. CLSM images were taken at 40 h after infiltration. Images present confocal projections composed of 20 optical sections taken at 1 µm intervals, from the surface to the middle of epidermal cells. Epidermal cells transiently expressing NbGAPDH-A-GFP and p27 fused with DsRed-monomer (p27-DRm). Green signals represent chloroplast-localizing NbGAPDH-A-GFP and red signals represent large aggregates formed by p27-DRm. No overlapping signals were detected. Scale bar = 20 µm.
(TIF)

Table S1 **LC/MS/MS analysis of proteins copurified with the tagged MP.** A piece of silver-stained gel below the 42 kDa marker (red arrow, Figure 2A) was subjected to LC/MS/MS analysis. RCNMV MP and several host proteins identified specifically to the tagged MP, not wild type MP.
(TIF)

Table S2 **List of the primers used in the study.**
(TIF)
