# Supplementary figures and images for: GAPDH-A Recruits a Plant Virus Movement Protein to Cortical Virus Replication Complexes to Facilitate Viral Cell-to-Cell Movement
Source: PLoS Pathog. 2014 Nov 20;10(11):e1004505. doi: 10.1371/journal.ppat.1004505 (PMC4239097; doi:10.1371/journal.ppat.1004505)

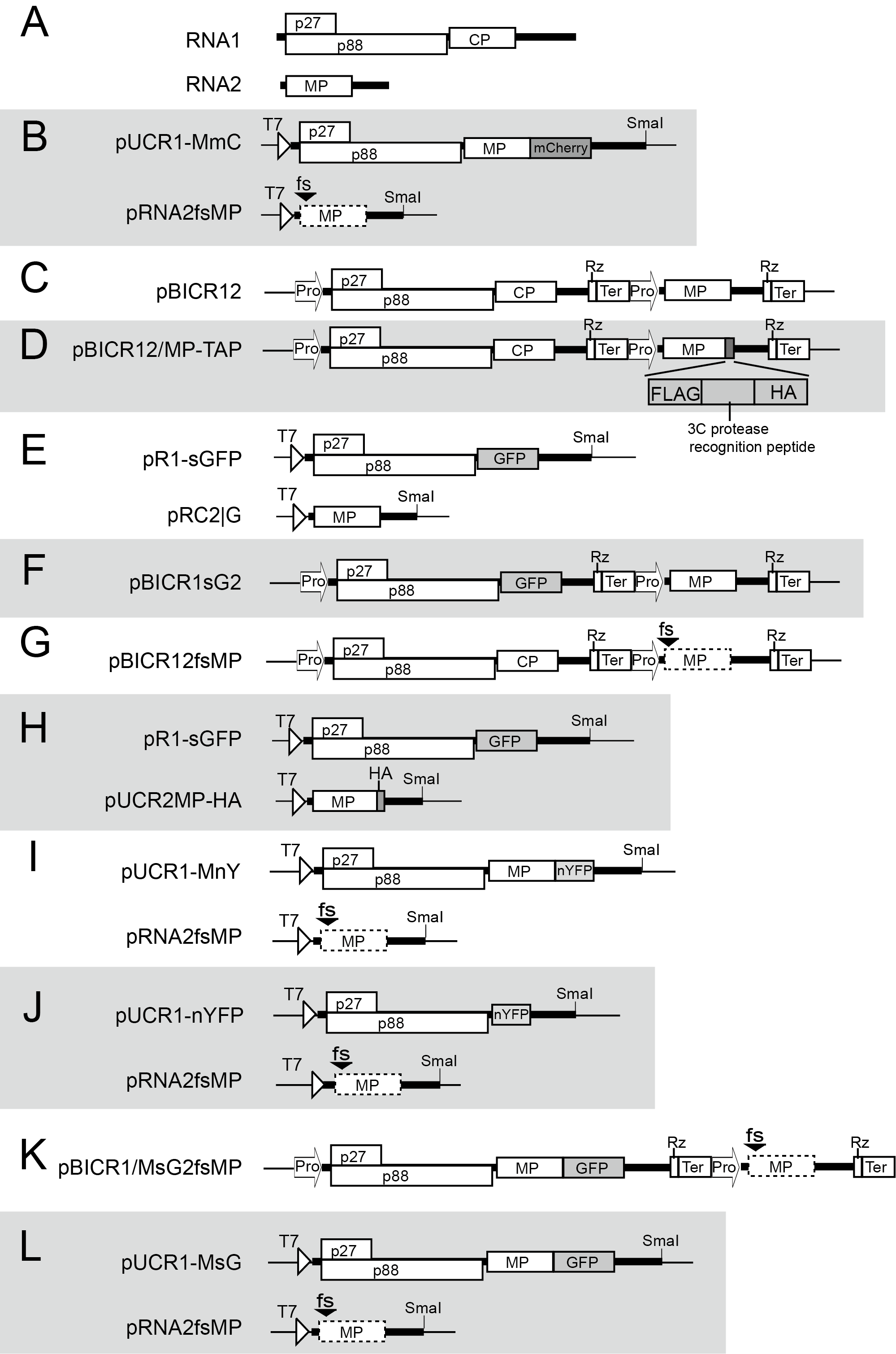

Supplement: Figure S1 — Schematic diagrams of Red clover necrotic mosaic virus (RCNMV) and various derivative constructs. (A) Genome map of RCNMV. Open boxes and bold lines show open reading frame (ORF) and the untranslated regions of the virus, respectively. (B–L) Plasmids containing the prefix ‘pUC’ and ‘pR’ and pRNA2fsMP were cut with SmaI and used as templates for in vitro transcription. Plasmids containing the prefix ‘pBIC’ were used for inoculation via Agrobacterium. Shaded boxes show the ORF of fluorescent proteins and tag peptides. Dashed boxes show the untranslated MP ORF; and fs is the four-nucleotide insertion for a frameshifting mutation. Key: T7, T7 promoter; Pro, Cauliflower mosaic virus (CaMV) 35S promoter; Ter, CaMV terminator; Rz, ribozyme sequence; SmaI, SmaI recognition sequence. (TIF) [file ppat.1004505.s001.tif]

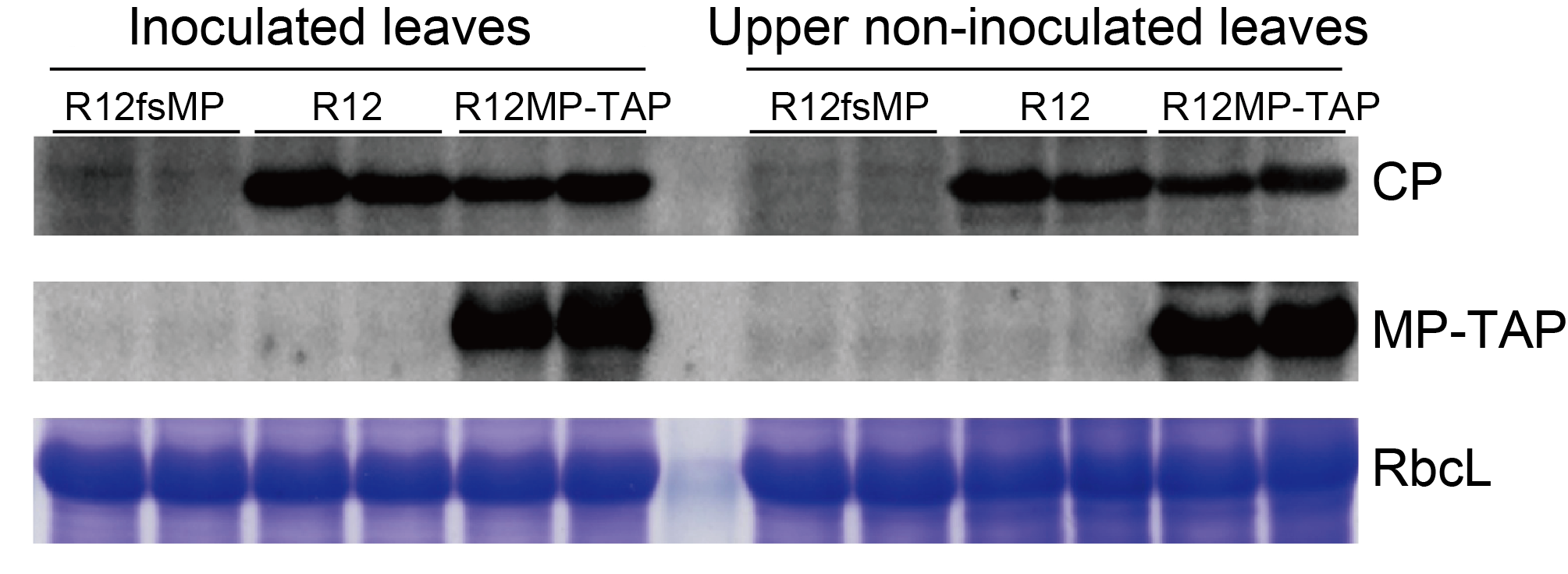

Supplement: Figure S2 — RCNMV MP fused with tandem affinity purification (TAP) tag sequence is functional. pBICR12 (Figure S1C) and pBICR12/MP-TAP (Figure S1D) and pBICR12fsMP (Figure S1G) [10] were inoculated to two young N. bethamiana plants via Agrobacterium using toothpicks (see ‘Virus-induced gene silencing’ paragraphs in Materials and Methods), respectively. Proteins were extracted from the inoculated leaves at 4 days post infiltration (dpi) and upper non-inoculated leaves at 7 dpi, respectively. 20 µg of samples was loaded to each lane. CP was detected using a rabbit polyclonal antibodies against RCNMV CP. MP-TAP was detected using a rat polyclonal antibodies against HA. RbcL is a Coomassie brilliant blue-stained gel image, which shows the large subunit of Rubisco proteins. (TIF) [file ppat.1004505.s002.tif]

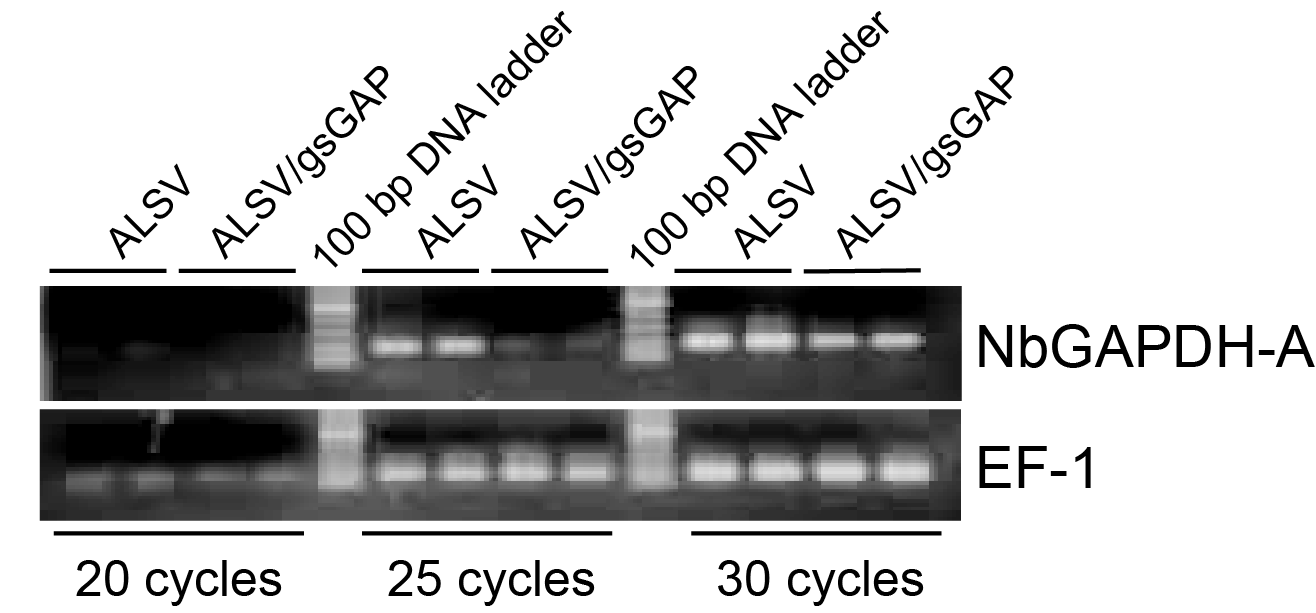

Supplement: Figure S3 — Semi-quantitative RT-PCR analysis of NbGAPDH-A mRNA accumulation levels in the ALSV vector-infected plants leaves. Total RNA was prepared from each of two independent plants inoculated with empty ALSV vector or ALSV/gsGAP vector. NbGAPDH-A mRNA levels were determined by semi-quantitative RT-PCR. The RT-PCR results for the EF-1 gene show that equal amounts of total RNA were used for RT, and the RT reaction had an equivalent efficiency with the samples. Primers used to amplify both genes are similar to those used in Figure 3A. (TIF) [file ppat.1004505.s003.tif]

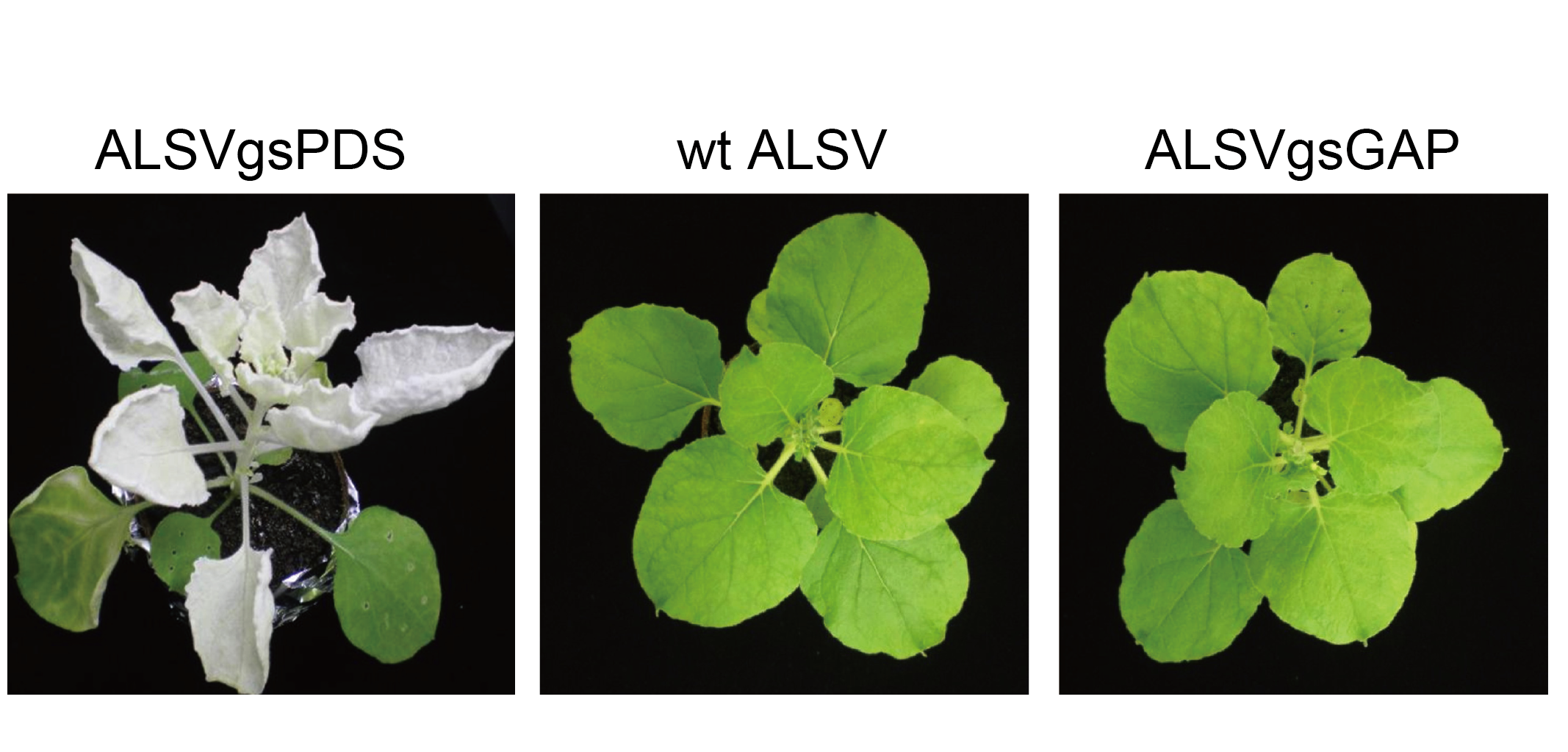

Supplement: Figure S4 — NbGAPDH-A -silenced plant as well as ALSV-infected plant does not exhibit any symptoms. Representative images of N. benthamiana plants 26 days post inoculation with ALSV vectors via Agrobacterium. N. benthamiana plants inoculated with the vector containing 102 nt of Phytoene desaturase (ALSVgsPDS) started to be white at 9 dpi. Infection with ALSV empty vector (wt ALSV) and the vector containing 294 nt of NbGAPDH-A gene (ALSVgsGAP) did not affect plant growth and no symptoms were detected. (TIF) [file ppat.1004505.s004.tif]

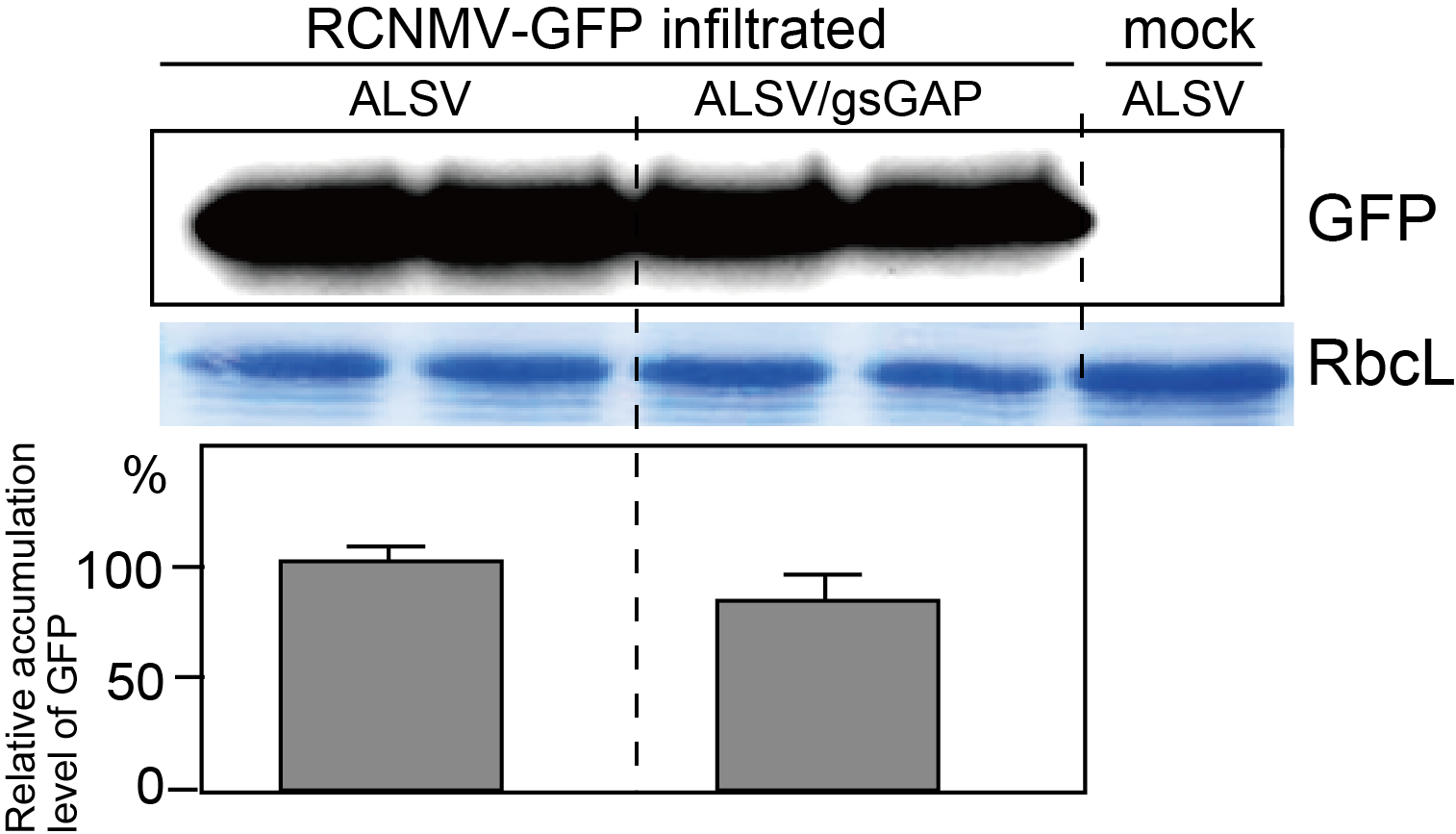

Supplement: Figure S5 — Multiplication of a recombinant RCNMV in NbGAPDH-A -silenced N. benthamiana leaves at a late stage of infection. An Agrobacterium culture that contained the pBICR1sG2 plasmid, which expressed RCNMV-GFP (Figure S1F), was diluted to OD600 = 0.03 and infiltrated into ALSV- or ALSV/gsGAP-infected N. benthamiana plants. At 48 hpi, protein was extracted from the infiltrated leaves and subjected to Western blotting using anti-GFP antibody. RbcL is a Coomassie brilliant blue-stained gel image, which shows the large subunit of Rubisco proteins. The accumulated levels of GFP from three separate experiments were quantified using the Image Gauge program and plotted in the graph. (TIF) [file ppat.1004505.s005.tif]

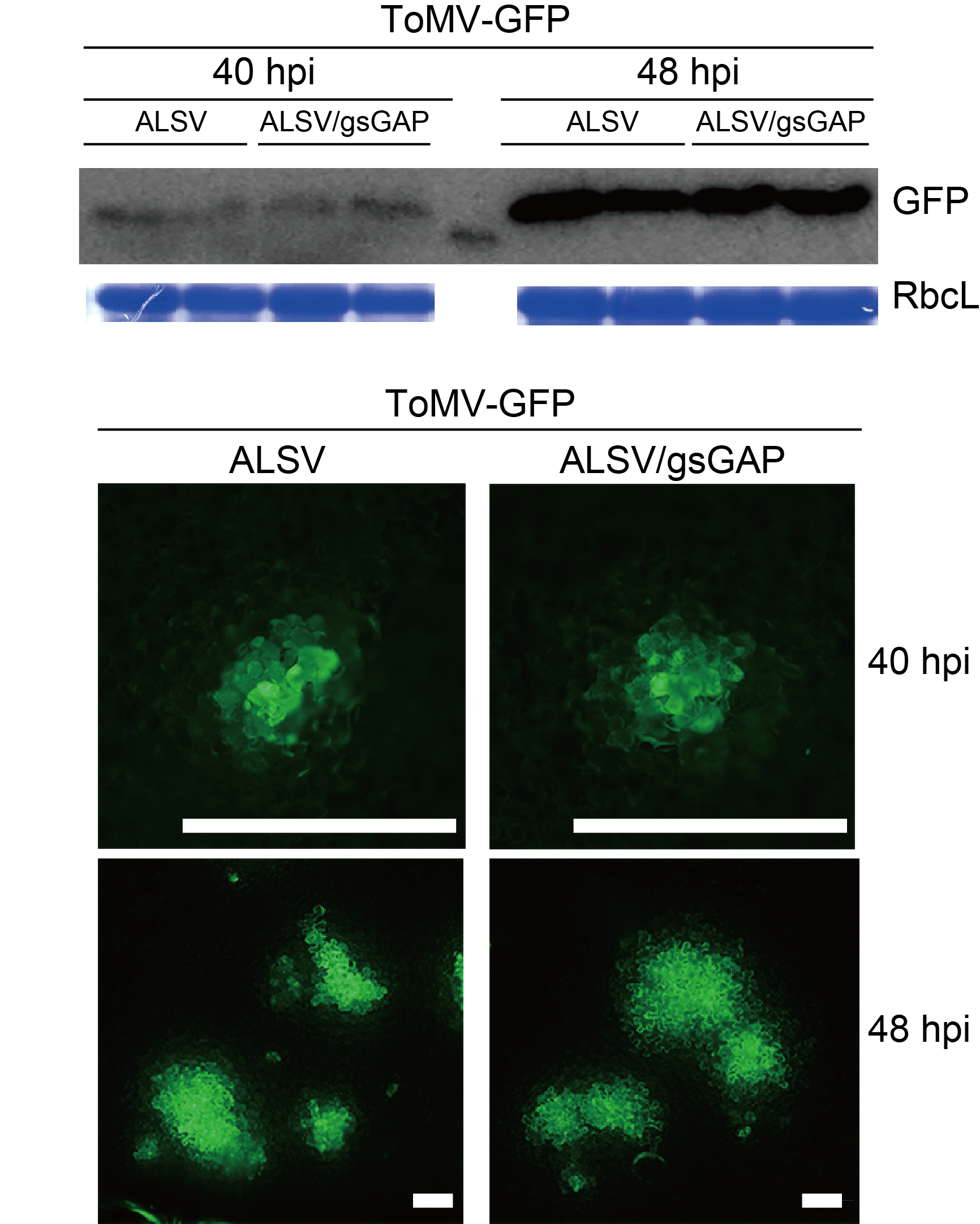

Supplement: Figure S6 — Multiplication of Tomato mosaic virus is not affected by the silencing of NbGAPDH-A . An Agrobacterium culture that contained the pToMVdCP-GFP plasmid, which expressed the recombinant Tomato mosaic virus in which the CP gene was replaced by GFP gene was diluted to OD600 = 0.03 and infiltrated into ALSV- or ALSV/gsGAP-infected N. benthamiana plants. At 40 and 48 hpi, protein was extracted from the infiltrated leaves and subjected to Western blotting using anti-GFP antibody. RbcL is a Coomassie brilliant blue-stained gel image, which shows the large subunit of Rubisco proteins. The lower panels are the representative epifluorescence microscopy images of the infiltrated leaves at 40 hpi and 48 hpi. Scale bar = 100 µm. (TIF) [file ppat.1004505.s006.tif]

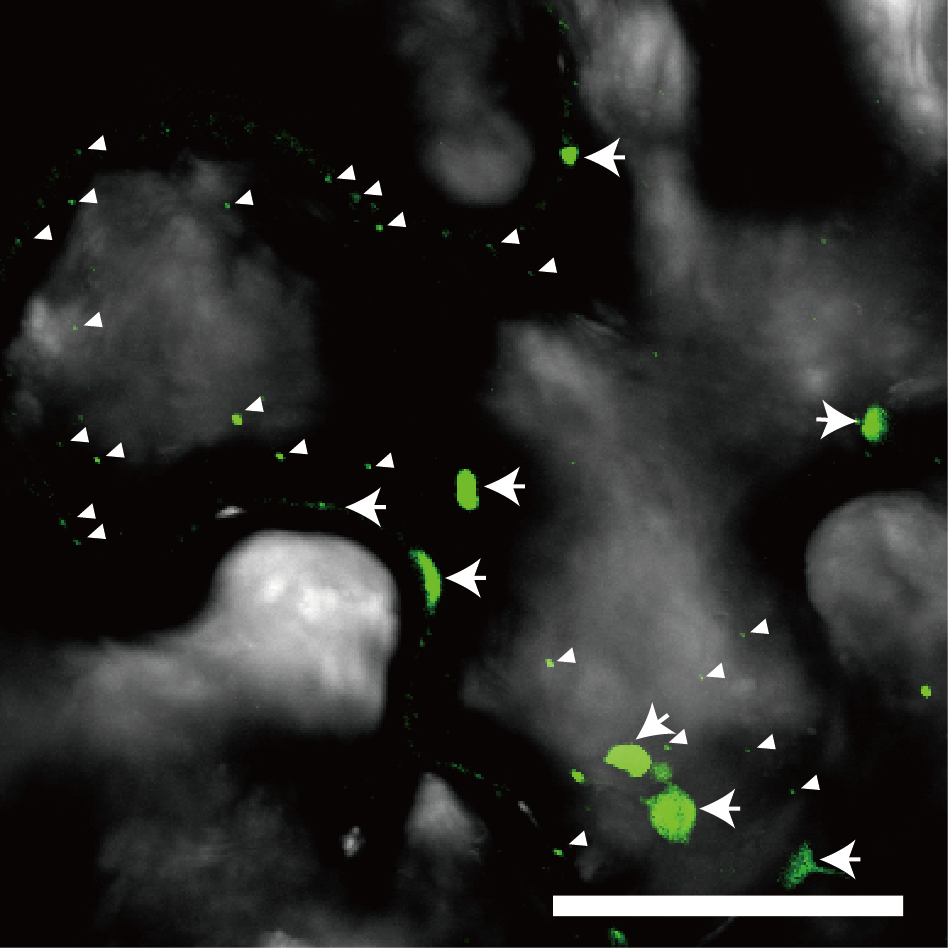

Supplement: Figure S7 — Subcellular localization of NbGAPDH-A-GFP coexpressed with RCNMV RNA1 and RNA2. Representative CLSM images of agroinfiltrated N. benthamiana epidermal cells which transiently expressed NbGAPDH-A-GFP with both RCNMV RNA1 and RNA2. Arrowheads represent cortical NbGAPDH-A-GFP signals and arrows represent chloroplast-localized NbGAPDH-A-GFP signal. Other conditions for infiltration and CLSM observation are similar to those in Figure 5. Scale bar = 20 µm. (TIF) [file ppat.1004505.s007.tif]

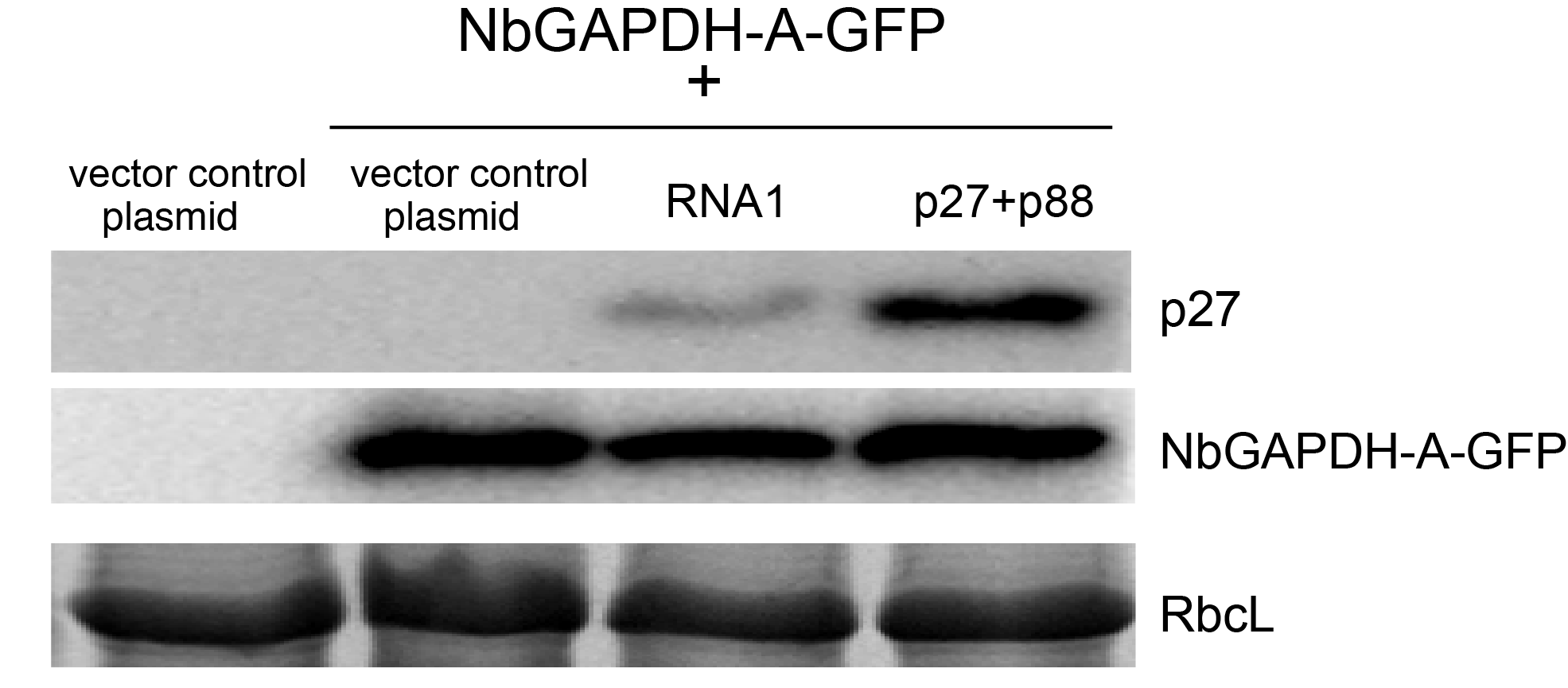

Supplement: Figure S8 — Accumulation of p27 and NbGAPDH-A in the agroinfiltrated leaves. NbGAPDH-A was expressed in N. benthamiana leaves together with RCNMV RNA1, or RCNMV replicase proteins p27 and p88. Each Agrobacterium culture was diluted to OD600 = 0.8 and equal volume of cultures were mixed and infiltrated into N. benthamiana leaves. At 40 hpi, protein was extracted from the infiltrated leaves and subjected to Western blotting using anti-p27 and anti-GFP antibodies. RbcL is a Coomassie brilliant blue-stained gel image, which shows the large subunit of Rubisco proteins. (TIF) [file ppat.1004505.s008.tif]

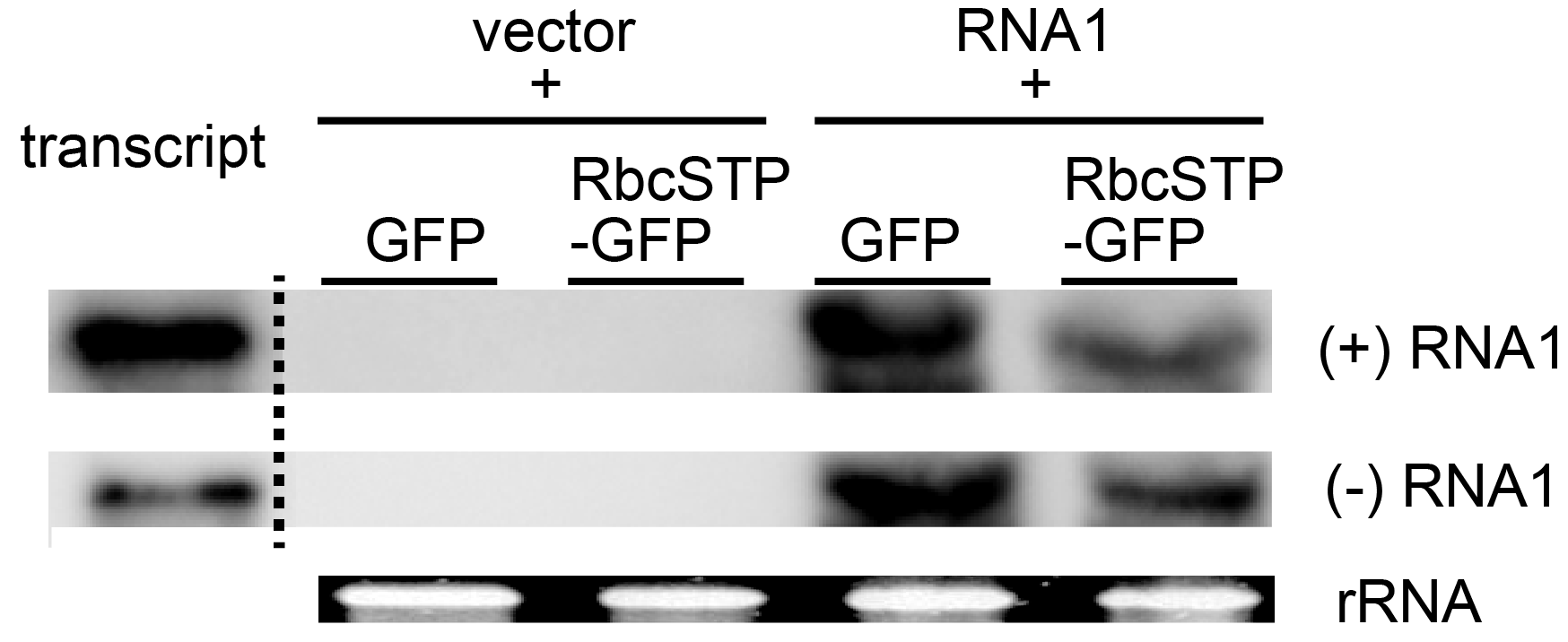

Supplement: Figure S9 — Accumulation of RNA1 in the agroinfiltrated leaves. An Agrobacterium culture that contained the plasmid that expressed RbcSTP-GFP, GFP, RCNMV RNA1 and that contained control vector plasmid was diluted to OD600 = 0.8. Equal volume of each combination of cultures was mixed and infiltrated into N. benthamiana plants. At 40 hpi, the total RNA was extracted from the infiltrated leaves and subjected to Northern blotting using DIG-labeled riboprobes specific for the plus (+)- and minus (−)-strand RNA1 of RCNMV. In vitro transcripts of (+)-RNA1 (10 µg) and (−)-RNA1 (1 µg) were loaded as the control marker. rRNA is an ethidium bromide-stained agarose gel image of 1 µg total RNA, which was used as the loading control. (TIF) [file ppat.1004505.s009.tif]

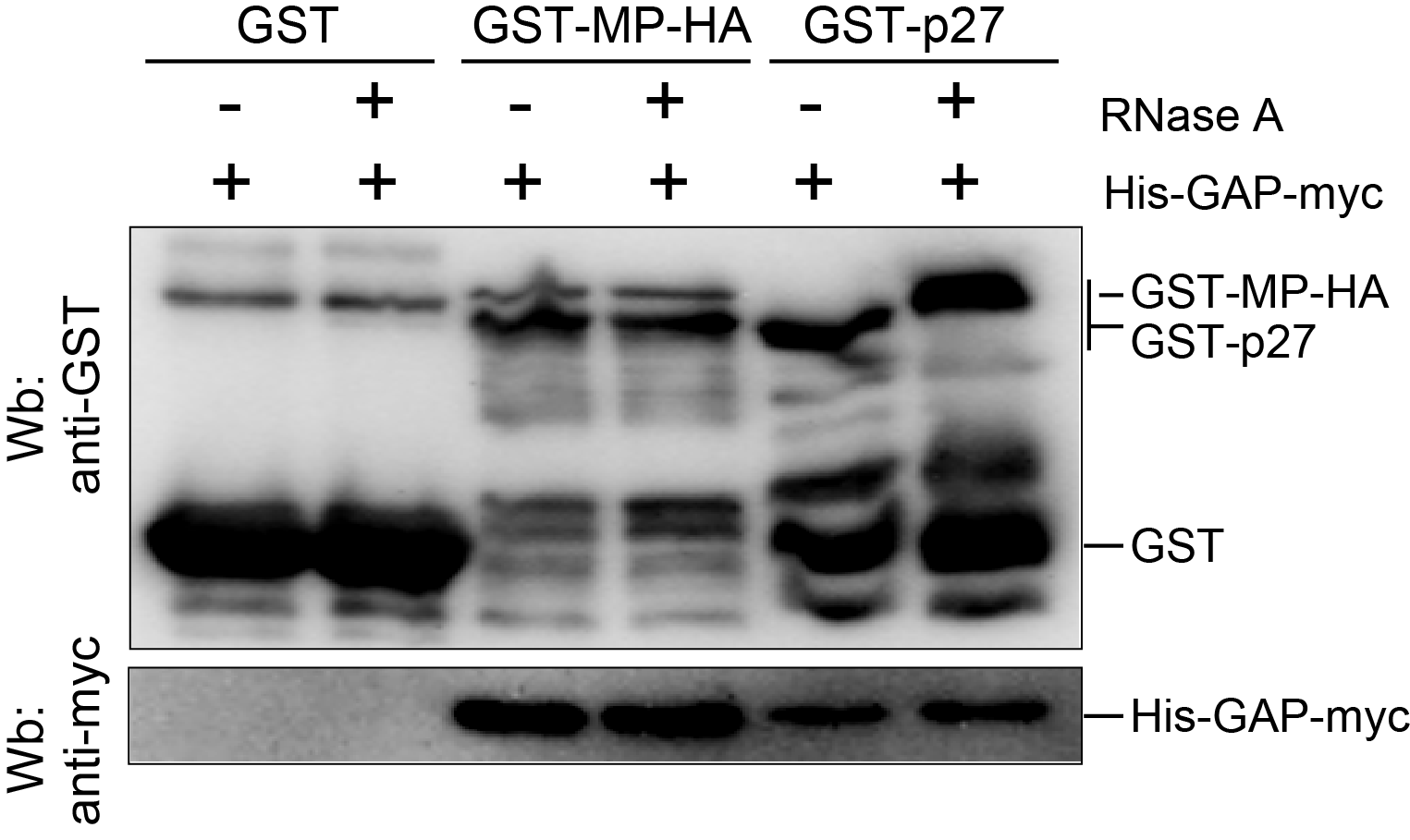

Supplement: Figure S10 — RNase treatment does not affect the interaction between NbGAPDH-A and RCNMV proteins in vitro . In the presence (+) or absence (−) of RNase A (50 µg/ml), glutathione resin-bound proteins were incubated with His-GAP-myc for 2 h at 4°C. The beads were then washed and the pulled-down complexes were subjected to SDS-PAGE and analyzed by Western blotting (Wb) using anti-GST and anti-myc antibodies. (TIF) [file ppat.1004505.s010.tif]

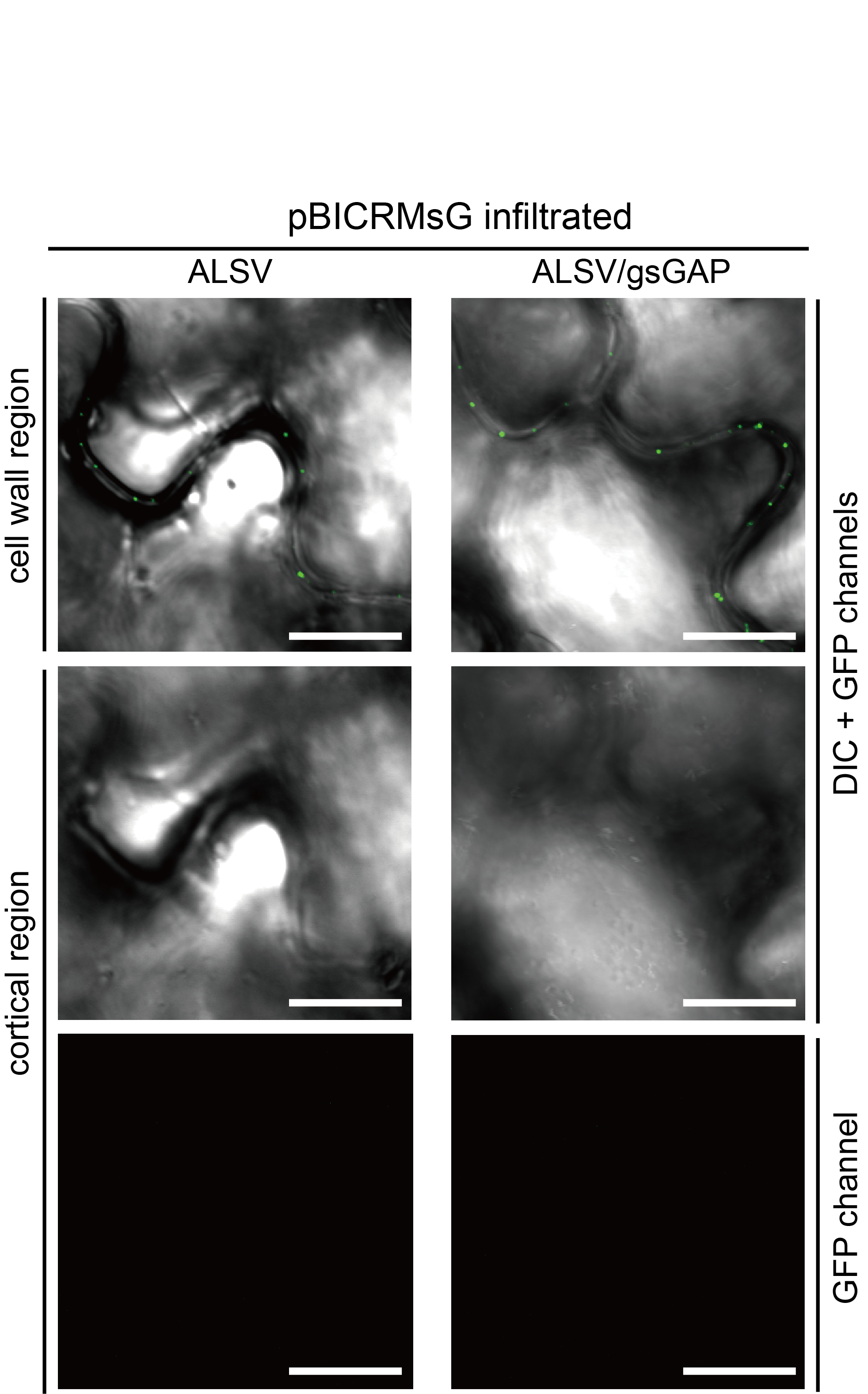

Supplement: Figure S11 — PD targeting of RCNMV MP-GFP is not affected by the silencing of NbGAPDH-A. Agrobacterium culture containing pBICRMsG plasmid that transiently expresses RCNMV MP-GFP under the control of Cauliflower Mosaic Virus 35S promoter [10] was diluted to OD600 = 0.8 and infiltrated into ALSV- or ALSV/gsGAP-infected N. benthamiana plants. Representative CLSM images of the leaves at 35 hpi show that the MP-GFP localized to the PD irrespective of the silencing of NbGAPDH-A. Scale bars = 20 µm. Images present confocal projections composed of 5 optical sections taken at 1 µm intervals, around cell wall region (top 2 panels) or cortical surface region (lower 4 panels) of epidermal cells. (TIF) [file ppat.1004505.s011.tif]

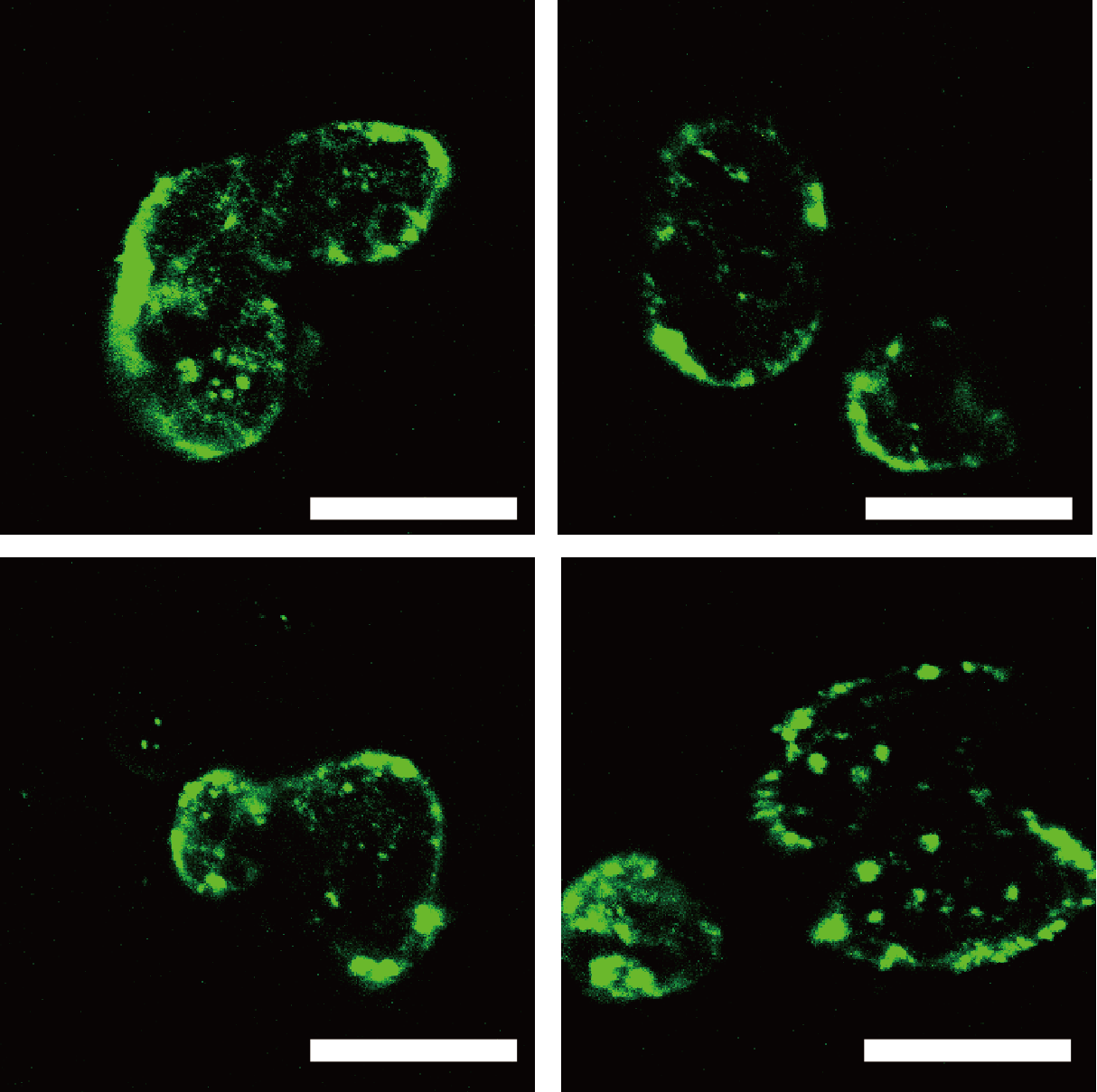

Supplement: Figure S12 — Localization of RCNMV MP-GFP in NbGAPDH-A –silenced plants at a later stage of infection of the recombinant virus that expresses MP-GFP. Representative CLSM images of ALSV/gsGAP-infected N. benthamiana mesophyll cells infiltrated with Agrobacterium cultures that contained pBICR1/MsG2fsMP, which expressed recombinant RCNMV RNAs encoding MP-GFP (Figure S1K). The images were obtained at 44 h after infiltration. Scale bar = 30 µm. The images represent confocal projections of 20 optical sections at 1 µm intervals, ranging from the surface to the middle of the cells. (TIF) [file ppat.1004505.s012.tif]

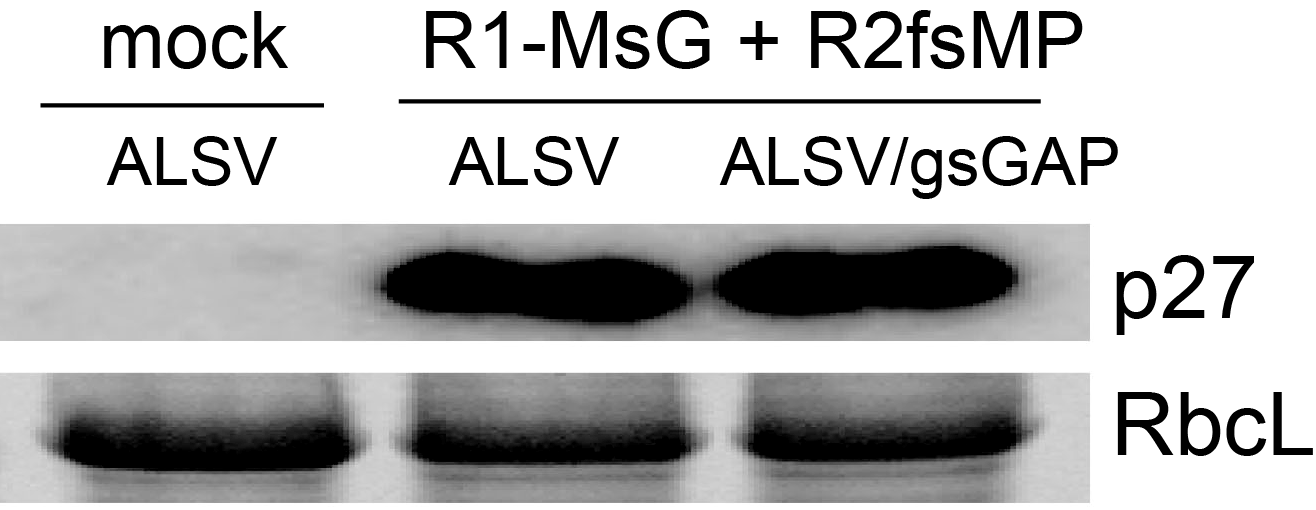

Supplement: Figure S13 — Accumulation of p27 in the protoplasts prepared from ALSV or ALSV/gsGAP-infected plants. Protoplasts prepared from ALSV- or ALSV/gsGAP-infected N. benthamiana plants were inoculated with recombinant RCNMV RNAs that expressed the MP-GFP fusion protein (Figure S1L). Protein was extracted Proteins extracted at 16 hpi from 2×10^4^ protoplasts were loaded in each lane. p27 was detected using the protein-specific rabbit polyclonal antibody. RbcL is a Coomassie brilliant blue-stained gel image of proteins extracted from 2×10^4^ protoplasts, which shows the large subunit of Rubisco proteins. The left-most panels show the results for mock-inoculated protoplasts treated with the same antibodies. (TIF) [file ppat.1004505.s013.tif]

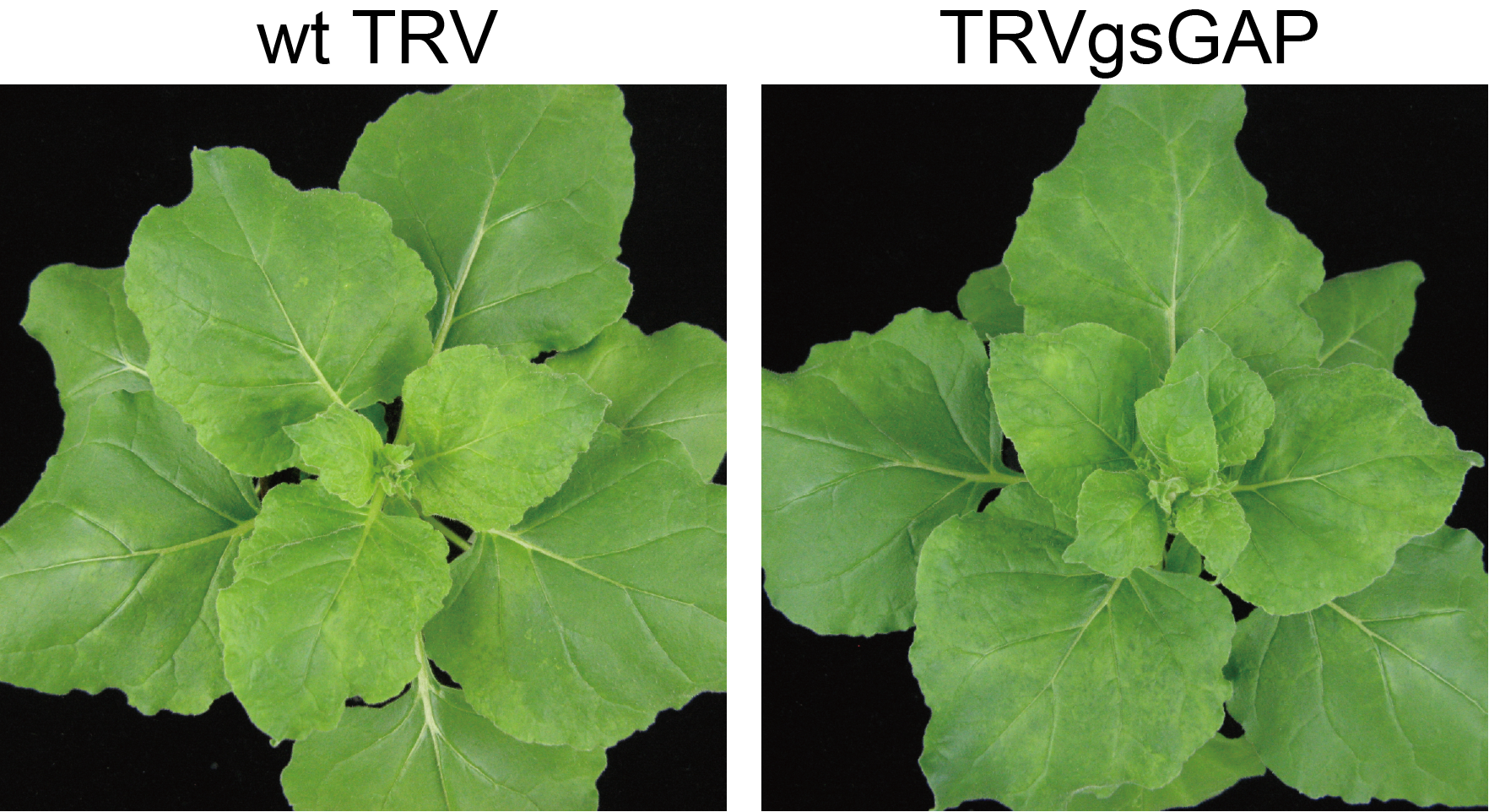

Supplement: Figure S14 — NbGAPDH-A -silenced plant by TRV-based vector exhibits similar mild symptoms as that by empty TRV vector-infected plant. Representative images of N. benthamiana plants 25 days post inoculation with TRV vectors via Agrobacterium. Infection with TRV empty vector (wt TRV) and the vector containing 294 nt of NbGAPDH-A gene (TRVgsGAP) did not affect plant growth and similar mild symptoms were detected. (TIF) [file ppat.1004505.s014.tif]

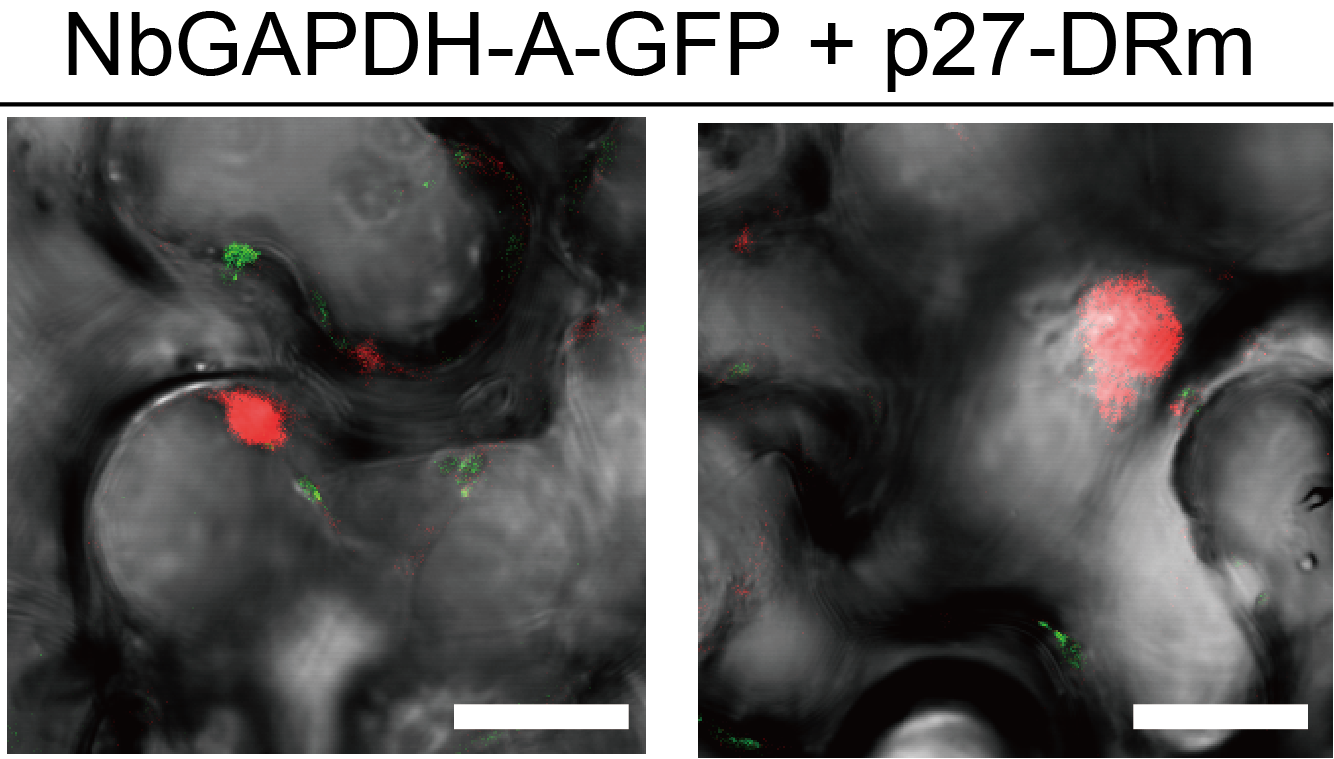

Supplement: Figure S15 — Subcellular localization of NbGAPDH-A-GFP is not affected by the coexpression of p27-DRm. Representative CLSM images of agroinfiltrated N. benthamiana cells. Each Agrobacterium culture was diluted to OD600 = 0.8 and equal volume of cultures were mixed for infiltration. CLSM images were taken at 40 h after infiltration. Images present confocal projections composed of 20 optical sections taken at 1 µm intervals, from the surface to the middle of epidermal cells. Epidermal cells transiently expressing NbGAPDH-A-GFP and p27 fused with DsRed-monomer (p27-DRm). Green signals represent chloroplast-localizing NbGAPDH-A-GFP and red signals represent large aggregates formed by p27-DRm. No overlapping signals were detected. Scale bar = 20 µm. (TIF) [file ppat.1004505.s015.tif]

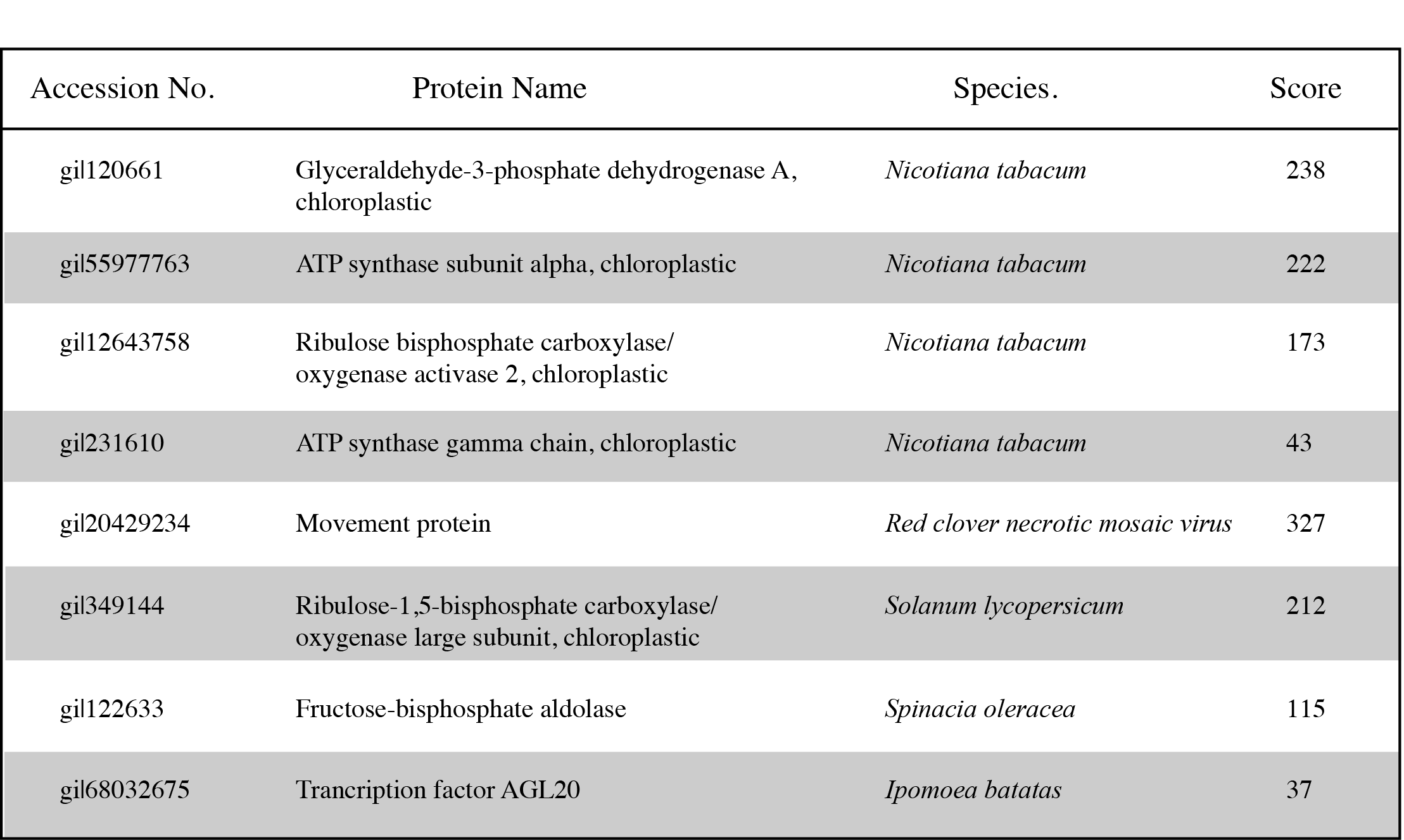

Supplement: Table S1 — LC/MS/MS analysis of proteins copurified with the tagged MP. A piece of silver-stained gel below the 42 kDa marker (red arrow, Figure 2A) was subjected to LC/MS/MS analysis. RCNMV MP and several host proteins identified specifically to the tagged MP, not wild type MP. (TIF) [file ppat.1004505.s016.tif]

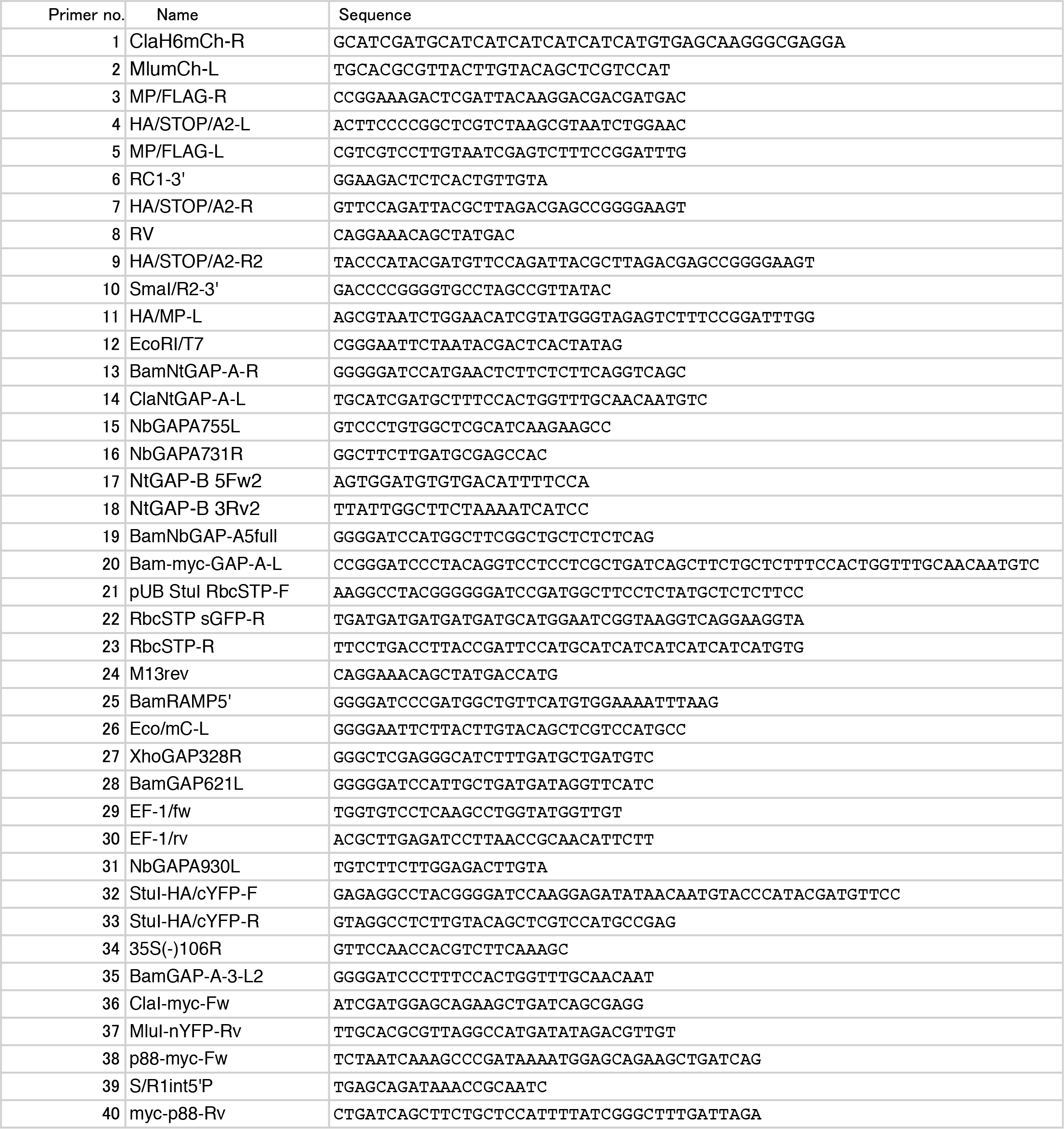

Supplement: Table S2 — List of the primers used in the study. (TIF) [file ppat.1004505.s017.tif]
